# In-silico evaluation of putative maternal semiochemicals of pigs with receptor proteins

**DOI:** 10.3389/fmolb.2025.1600209

**Published:** 2025-08-08

**Authors:** Devaraj Sankarganesh, Ambritha Balasundaram, Hayavadhan Sampath, Diya Manjunath, George Priya Doss C.

**Affiliations:** School of BioSciences and Technology, Vellore Institute of Technology (VIT), Vellore, Tamilnadu, India

**Keywords:** pheromones, pig farming, olfaction, vomeronasal organ, main olfactory system

## Abstract

Piglets at weaning experience stress owing to environmental changes. Mixing unfamiliar littermates also induces fighting and biting behaviors among them, affecting their welfare. In addition, post-weaning weight gain or loss is also influenced during the first week of weaning. Many compounds have been identified in the secretions of sows to address these behavioral and welfare issues; nevertheless, the positive influence of these compounds on piglet behavior and welfare is not fully understood. Therefore, we sought to study the interaction between the compounds (myristic acid, oleic acid, lauric acid, palmitic acid, 3-methyl phenol, tiglic aldehyde, and skatole, reported as maternal pheromones/urinary metabolites) and receptor proteins using computational approaches. We used five proteins, including alpha-1-acid glycoprotein (AGP), odorant binding protein (OBP), salivary lipocalin (SAL), pheromaxein, and Von Ebner’s Gland Protein (VEGP). We utilized molecular docking with AutoDock Vina and molecular dynamics simulations (MDS) using GROMACS to examine the stability of interactions between the listed compounds and proteins. The binding energies for the docked complexes ranged between −3.4 and −6.7 kcal/mol. Through analysis of the lowest root mean square deviation (RMSD) and hydrogen bond formations, we identified that at least one of the fatty acids exhibited optimal docking with four distinct proteins. The RMSD data for these complexes also indicated stability over a 100-ns MDS period. However, the post-MDS Molecular Mechanics/Poisson Boltzmann Surface Area (MM/PBSA) binding energy data revealed that palmitic acid had the highest stabilizing energy across all five proteins compared to other complexes. Additionally, myristic acid and oleic acid also exhibited a high binding affinity with the proteins. Taken together, our findings suggest that fatty acids could be the most effective semiochemicals for managing behavioral and welfare issues in weaning piglets.

## Introduction

Pheromones are species-specific chemical signals secreted into body fluids, such as urine, feces, saliva, and glandular secretions. Pheromones trigger both short- and long-term behavioral and neuroendocrinological changes in conspecifics (Tirindelli et al., 2009); therefore, synthetic analogs of pheromones have been used in the management of behavioral problems in animals. Pigs rely heavily on pheromones for social communication. The introduction of a boar to a group of prepubertal gilts induced LH pulse frequency, implying the importance of boar-derived pheromones and their positive influence on endocrine changes (Kingsbury and Rawlings, 1993). Indeed, the presence of steroid pheromones (androstenone and androstenol) has been confirmed in boar saliva (Patterson, 1968). These salivary steroid pheromones, in combination with quinoline, induced a high sexual behavior score in sows (McGlone et al., 2017). However, the existence of quinoline was not confirmed in boar saliva (Sankarganesh et al., 2021). It was also observed that the individual compound in the mixture elicited specific behaviors. When combined, the mixture of three compounds (steroid pheromones and quinoline) elicited a high incidence of sexual behaviors in the gilts and sows, implying that each compound in the mix contributes to the specific odor of the conspecifics. In addition to its positive influence on sexual communication, androstenone also reduced aggressive behavior in regrouped piglets at weaning and improved the average daily gain and gain-to-feed ratio (McGlone et al., 1986; McGlone and Morrow, 1988).

The reduction of weaning stress in piglets is pivotal, as it leads to productivity loss by reducing immunity and increasing plasma corticosterone levels (Moore et al., 1994). There were also significant shifts in aerodigestive coordination before and after weaning, reflecting developmental changes that may contribute to behavioral adaptability during this transitional phase (Bond et al., 2020). Weaning also promotes fighting behaviors and increases aggression, lesions, and weight loss in piglets (Mei et al., 2016). Therefore, developing a solution to mitigate behavioral problems in weaning piglets is obligatory. In this sense, pheromones identified in the mammary gland area have been proposed to mitigate behavioral problems at weaning. These molecules were identified as a mixture of fatty acids, including linoleic, oleic, and myristic acids (Pageat, 1998). These fatty acids have been extensively studied for their ability to reduce agonistic behaviors, and their application has been found to improve welfare in various pig breeds and situations (Guy et al., 2009; Yonezawa et al., 2009; Marcet-Rius et al., 2022).

As weaning issues in piglets are highly notorious, the search for molecules has been on the rise. For instance, McGlone et al. (2017) utilized the maternal pheromone of rabbits (2-methyl-2-butenal; 2M2B) to alleviate weaning-associated behavioral problems in piglets. They found increased feed intake during the first 24 h after applying 2M2B on the feeder, in addition to an increase in the average daily gain in the piglets. The feces of lactating sows suggest that olfactory cues play a significant role (Horrell and Hodgson, 1992; Morrow-Tesch and McGlone, 1990). Subsequently, Aviles-Rosa et al., 2020 reported skatole and myristic acid in the feces of farrowing sows, which were believed to modify the behavior of weaning piglets. They also showed that when these molecules were supplied as a mixture on the feeder of weaned piglets, the fighting and biting behaviors were significantly reduced. It is worth noting that myristic acid, identified in the feces, was also reported in the fatty acid mixture of pig appeasing pheromones (Pageat, 1998). Molecules with related properties have also been identified in the secretions of immune system-stimulated pigs. In particular, 3-methylphenol and 4-ethylphenol have been identified in increased concentrations in the immune system-stimulated pigs, which, when tested with piglets, induced repulsion behaviors (Devaraj et al., 2019).

Although many molecules have been shown to modify the behavior of piglets, studies on the olfactory effects of these molecules are scarce. It also necessitates the evaluation of the binding efficacy of the molecules with the olfactory receptor proteins of pigs. In pigs, five different proteins play key roles in the reception of volatile signals arising from the external environment (Sankarganesh et al., 2022). Odorant-binding protein (OBP), found primarily in the nasal epithelium of pigs, binds to a range of odorants, including fatty acids and steroids. Salivary lipocalin (SAL) is of submaxillary gland origin in boars, which binds to sex pheromones such as androstenone and androstenol and plays a critical role in transporting pheromones to the olfactory sensory neurons, thereby facilitating sexual communication. SAL isoforms are found in the nasal mucosa and the VNO, each with unique binding affinities for steroid pheromones. Pheromaxein is a 16-androstene steroid-binding protein, predominantly synthesized in the submaxillary glands of pigs (Austin et al., 2004). Von Ebner’s gland protein (VEGP) is present in the nasal epithelium of pigs. In addition to its high binding affinity for fatty acids, such as palmitic and oleic acids, VEGP also exhibits a high binding affinity for progesterone, suggesting its role in pheromone signaling and communication. Alpha-1-acid glycoprotein (AGP) in pigs is synthesized in the liver, and its levels vary according to age, sex, and health status (Guiraudie et al., 2003). Together, these proteins facilitate crucial aspects of chemical communication, stress responses, and pheromone detection in pigs.

In this purview, testing the binding efficacy of these proteins with different molecules would be pertinent. This would give us an overview of the possibility of the molecules interacting with the proteins. It would also help us obtain an appropriate mixture of molecules to modify piglet behavior at weaning and any other similar stressful events.

## Methods

### Preparation of protein structures

High-resolution crystallized odorant binding protein (OBP) with the PDB ID:1DZK and salivary lipocalin (SAL) with the PDB ID:1GM6 structures in 3-D were obtained from the RCSB-PDB. The 3-D structures of Pheromaxein (AF-Q863D3-F1) and Von Ebner’s Gland Protein (VEGP) (AF-P53715-F1) were retrieved from the AlphaFold Protein Structure Database because of the unavailability of the structures (Sankarganesh et al., 2024). The coding sequence of Alpha-1-acid glycoprotein (AGP) was obtained from NCBI-Genbank. The three-dimensional (3D) structure was generated using the I-TASSER server, as no 3D crystalline PDB structure was available on the Research Collaboratory for Structural Bioinformatics Protein Data Bank (RCSB PDB) site (Kouranov et al., 2006; Roy et al., 2010; Sayers et al., 2022). The modeled structure validation was done using SAVES-PROCHECK to assess the structural integrity (Laskowski et al., 1993). PyMOL was used to visualize the protein structures, clean the protein, add polar hydrogens, and save the structure in. pdb format (Lill and Danielson, 2011). We utilized the Swiss-PDBViewer’s GROMOS96 tool to minimize the energy of all protein structures (Kaplan and Littlejohn, 2001). The protein structure was then converted into a PDBQT file. The format was modified after AutoDock tools added polar hydrogen, Kollman charges, Gasteiger charges, and assigned AD4-type atoms (Morris et al., 2009).

### Selection and preparation of ligands for molecular docking

The ligands were chosen from the literature analysis of compounds involved in sow secretions and the biological properties of the identified compounds to address the behavioral and welfare concerns of weaning piglets. We used 3-methylphenol, four fatty acids (lauric, myristic, oleic, and palmitic acids), skatole (methylindole), and tiglic aldehyde. The Two-Dimensional (2D) structures of all the ligands were retrieved (in PNG format) from PubChem and included in Supplementary Table S1. The downloaded Three-Dimensional (3D) structures of these compounds (SDF format) from PubChem were converted into pdb structures using the BIOVIA Discovery Studio Visualizer (Biovia, 2017; Kim et al., 2023). Structural minimization of the compounds was performed using Avogadro software’s Merck Molecular Force Field (MMFF94) (Hanwell et al., 2012). The conversion from pdb format to pdbqt format was achieved using AutoDock Tools, where each ligand was individually directed to Torsion Tree, which utilized Detect Root to determine the torsion center axis, and then configured torsional degrees within Torsion Tree; Gasteiger partial charges were assigned during this process (Morris et al., 2009).

### Molecular docking

We used the Computed Atlas of Surface Topography of Proteins (CASTp) server 3.0 to identify the active sites of the proteins (Binkowski et al., 2003). We ensured that the grid box of the receptor was positioned within the active site region of the protein. The predicted key residues from the CASTp server, as well as the 3D structure and active site of each protein, were listed in the Supplementary Table S2. AutoDock Vina-aided molecular docking was used to predict ligand conformations within the orthosteric pocket of the target protein, with ten postures, four energy ranges, and a 32-fold exhaustiveness (Trott and Olson, 2010). The docking simulation was carried out using grid box settings centered on the active region of the protein binding pocket. Supplementary Table S3 provides the grid box’s coordinates and dimensions parameters for all protein structures utilized in docking studies. The exhaustiveness parameter was set to 16 to provide appropriate conformational sampling with 10 postures and four energy ranges. AutoDock Vina’s search technique used a dynamic global optimization approach to efficiently investigate various binding modes. The optimal protein-ligand complex was identified based on its binding energy (kcal/mol). Maestro Schrödinger Suite 2021 was used to visualize protein-ligand interactions in two dimensions (2D), where the orientation with the least RMSD and the most favorable binding energy was further chosen for the molecular dynamics simulation (Firdhouse and Lalitha, 2015).

### Molecular dynamics and simulation

Molecular Dynamics Simulations (MDS) with GROMACS software (v. 2021.2) were used to study protein-ligand complexes (Abraham et al., 2015). For both the ligand and receptor, the Charmm27 force field was obtained from the SwissParam website (Brooks et al., 2009; Zoete et al., 2011). Using the TIP3P water model, the systems were solved in a triclinic box and neutralized with an isotonic NaCl solution. Long-range electrostatics were computed using the Particle Mesh Ewald (PME) method, with a Van der Waals cut-off of 1.0 nm and short-range electrostatics applied. The LINCS algorithm was used to constrain hydrogen covalent bonds, allowing for a 2-fs time step. The steepest descent algorithm was used to minimize energy. Two-stage NVT and NPT equilibria were established for 100 ps each. The V-rescale thermostat was used to maintain a temperature of 300 K, and the Parrinello-Rahman barostat was used to support a pressure of 1 bar. After equilibration, the production MD simulation was executed for 100 ns with periodic boundary conditions. Post-simulation, using gmx rms, gmx rmsf, and gmx hbond, root mean square deviation (RMSD), root mean square fluctuation (RMSF), and the number of hydrogen bonds were computed to evaluate stability, flexibility of the structure over the 100 ns, and to analyse the H-bond interactions between the proteins and ligands. The calculation of binding free energies of the complexes was done using the Molecular Mechanics/Poisson Boltzmann Surface Area (MM/PBSA) technique, which incorporates the Poisson Boltzmann solvation model. The equilibrated portion of the MDS trajectory was used for this computation (Kumari et al., 2014). Further, we estimated the binding free energy over the equilibrated portion of MDS trajectory. The binding affinity of two reactants combined at constant temperature and pressure is defined by the change in Gibbs free energy, also known as binding free energy, ΔG = ΔH–TΔS where T = temperature (K), ΔS = the change in entropy (J/mol K), and ΔH the change in enthalpy (kJ/mol) (Wright et al., 2014).

## Results

### Protein structure validation and active site region prediction

Prior to molecular docking, the structure of Alpha-1-acid glycoprotein was validated using Ramachandran plot from SAVES-PROCHECK. We found that 98.1% of the residues were in the most favorable and allowed regions (Supplementary Figure S1). The active site of Alpha-1-acid glycoprotein contains TYR25, SER28, PHE30, GLN34, TYR35, SER38, ALA39, ILE42, ALA45, PHE47, PHE49, LEU60, GLU62, GLN64, ASN73, SER75, SER76, LEU77, LEU86, SER87, LYS88, HIS89, GLU90, ARG93, HIS95, ALA97, LEU110, ASN112, GLY121, SER123, PHE124, and TYR125, were predicted using CASTp. The active sites of other proteins, such as OBP, SAL, Pheromaxein, and VEGP, were acquired using the CASTp server, as described previously (Supplementary Table S2) (Sankarganesh et al., 2024).

### Molecular docking analysis reveals the ligand binding energy for the different proteins

**FIGURE 1:**
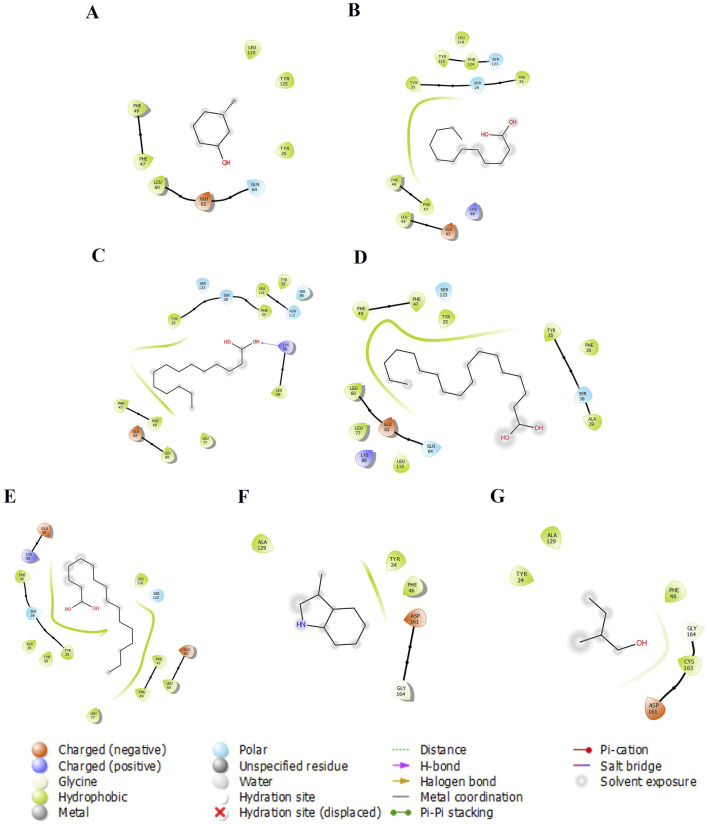
The 2-D molecular interaction of the best docking poses of the compounds with AGP. **(A)** AGP-3-Methylphenol, **(B)** AGP-Lauric Acid, **(C)** AGP-Myristic Acid, **(D)** AGP-Oleic Acid, **(E)** AGP-Palmitic acid, **(F)** AGP-Skatole, and **(G)** AGP-Tiglic aldehyde.

**FIGURE 2:**
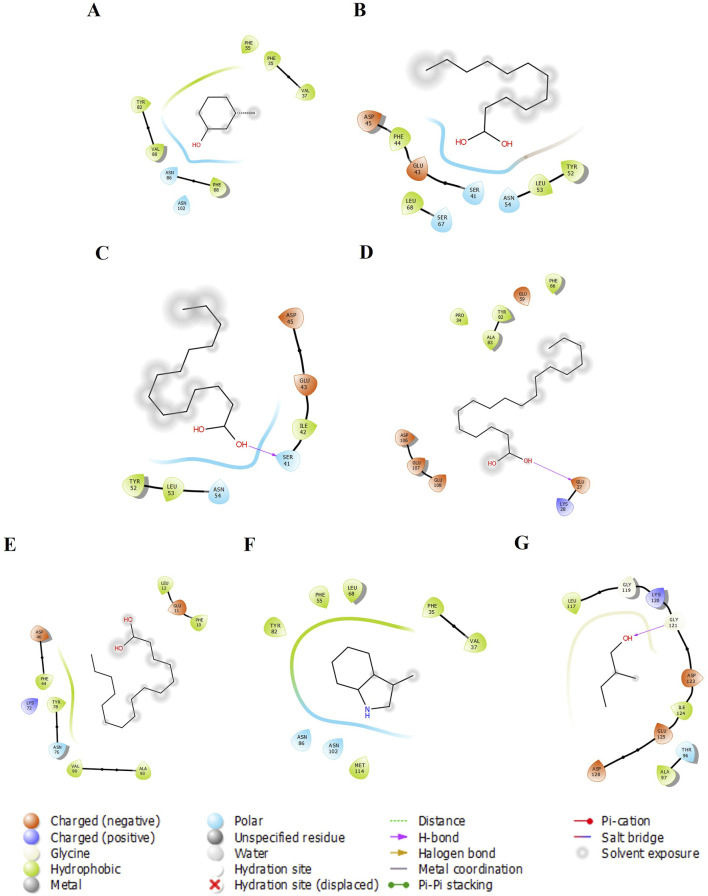
The 2-D molecular interaction of the best docking poses of the compounds with OBP. **(A)** OBP-3-Methylphenol, **(B)** OBP-Lauric Acid, **(C)** OBP-Myristic Acid, **(D)** OBP-Oleic Acid, **(E)** OBP-Palmitic Acid, **(F)** OBP-Skatole, and **(G)** OBP-Tiglic aldehyde.

**FIGURE 3:**
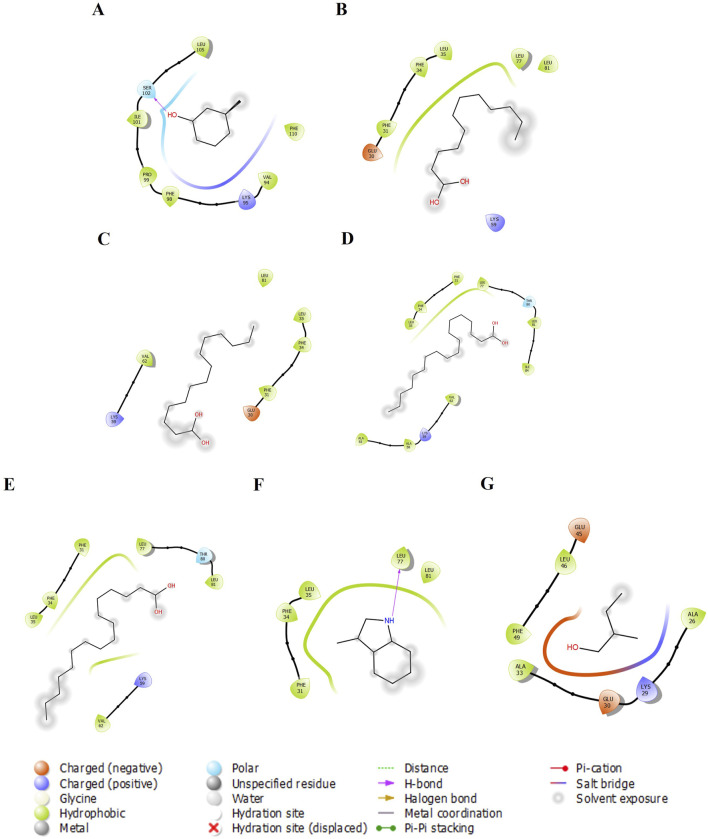
The 2-D molecular interaction of the best docking poses of the compounds with Pheromaxein. **(A)** Pheromaxein-3-Methylphenol, **(B)** Pheromaxein-Lauric Acid, **(C)** Pheromaxein-Myristic Acid, **(D)** Pheromaxein-Oleic Acid, **(E)** Pheromaxein-Palmitic Acid, **(F)** Pheromaxein-Skatole, and **(G)** Pheromaxein-Tiglic aldehyde.

**FIGURE 4:**
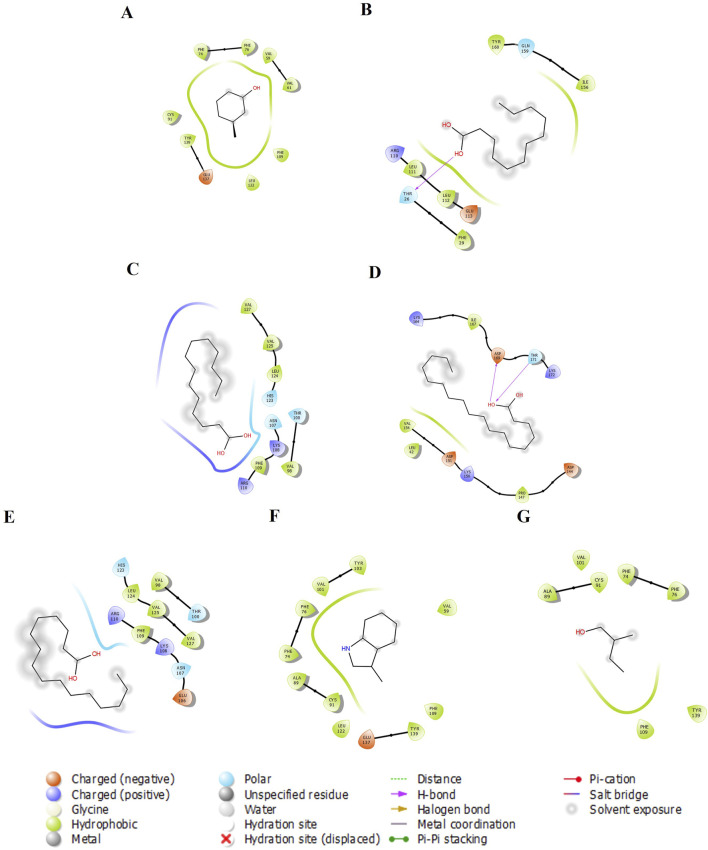
The 2-D molecular interaction of the best docking poses of the compounds with SAL. **(A)** SAL-3-Methylphenol, **(B)** SAL-Lauric Acid, **(C)** SAL-Myristic Acid, **(D)** SAL-Oleic Acid, **(E)** SAL-Palmitic Acid, **(F)** SAL-Skatole, and **(G)** SAL-Tiglic aldehyde.

**FIGURE 5:**
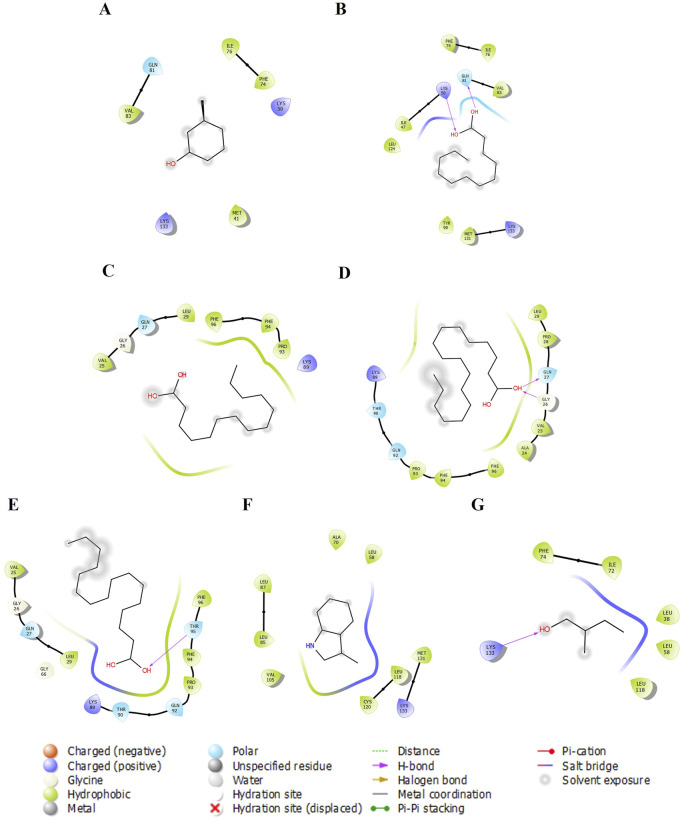
The 2-D molecular interaction of the best docking poses of the compounds with VEGP. **(A)** VEGP-3-Methylphenol, **(B)** VEGP-Lauric Acid, **(C)** VEGP-Myristic Acid, **(D)** VEGP-Oleic Acid, **(E)** VEGP-Palmitic Acid, **(F)** VEGP-Skatole, and **(G)** VEGP-Tiglic aldehyde.

We examined five proteins, including AGP, OBP, SAL, Pheromaxein, and VEGP, against seven compounds, including 3-methylphenol, four fatty acids (lauric, myristic, oleic, and palmitic acids), skatole (methylindole), and tiglic aldehyde. The binding energies of 35 complexes ranged from −3.4 to −6.7 kcal/mol (Table 1). Figures 1–5 depict the 2-D representation of the protein-ligand complexes. AGP complexes with 3-methylphenol, myristic acid, oleic acid, and skatole revealed more negative binding energies (<−5.4 kcal/mol) than the other complexes. AGP complexes with lauric acid, myristic acid, oleic acid, and palmitic acid had more interacting residues (12–14); among these, myristic acid formed a hydrogen bond with LYS88 (Table 1; Figure 1). 3-methylphenol and skatole showed more negative binding energies with OBP and SAL (<−5.4 kcal/mol), while oleic acid had a more negative binding energy (<−5.4 kcal/mol) with pheromaxein than other compounds. OBP complexes with lauric acid, oleic acid, palmitic acid, and tiglic aldehyde had more interacting residues (9–10 residues); nevertheless, myristic acid formed a hydrogen bond with SER41, oleic acid formed a hydrogen bond with GLU27, and tiglic aldehyde formed a hydrogen bond with GLY121 (Table 1; Figure 2). In Pheromaxein complexes, palmitic acid and oleic acid had the most interacting residues (8–11 residues), whereas 3-methylphenol and skatole established hydrogen interactions with SER102 and LEU77, respectively (Table 1; Figure 3). In SAL complexes, myristic acid, palmitic acid, and skatole had the most interacting residues (10–11 residues), but only oleic acid formed hydrogen interactions with ASP169 and THR171 (Table 1; Figure 4). With VEGP, skatole showed a more negative binding energy (−5.4 kcal/mol) compared to other compounds. In VEGP complexes, oleic acid, palmitic acid, and skatole had the most interacting residues (9–11 residues), with oleic acid forming hydrogen bonds with residues GLY26 and GLN27 and palmitic acid forming a hydrogen interaction with THR95. Lauric acid, on the other hand, formed hydrogen bonds with residues LYS50 and GLN81, as well as with eight more interacting residues (Table 1; Figure 5). Binding affinities confirmed the firm docking of the compounds and their appropriate orientation in the active sites of the proteins.

**TABLE 1:** Molecular docking results of various complexes with the binding energy values, interacting hydrogen bond residues, and other interacting residues.

Protein	Compounds	Binding energy (Kcal/mol)	Interacting residues beyond hydrogen bonds	Interacting hydrogen bond residues
AGP	3-Methylphenol	−5.5	8 (TYR25, PHE47, PHE49, LEU60, GLU62, GLN64, LEU110, TYR125)	None
	Lauric Acid	−5.2	12 (TYR25, SER28, PHE30, PHE47, PHE49. LEU60, GLU62, LYS88, LEU110, PHE124, TYR125, SER123)	None
	Myristic Acid	−5.4	14 (TYR25, SER28, PHE30, TYR35, PHE47. PHE49, GLU62, LEU60, LEU77, LEU86, HIS95, LEU110, ASN112, SER123)	1 (LYS88)
	Oleic Acid	−5.5	14 (TYR25, PHE30, TYR35, SER38, ALA39, PHE47, PHE49, LEU60, GLU62, GLN64, LEU77, LYS88, LEU110, SER123)	None
	Palmitic Acid	−5.2	14 (TYR25, SER28, PHE30, ALA39, TYR35, PHE47, PHE49, LEU60, GLU62, LYS88, LEU77, GLU90, LEU110, SER123)	None
	Skatole	−5.7	4 (TYR24, PHE46, ASP161, GLY164)	None
	Tiglic aldehyde	−4	6 (TYR24, PHE46, ALA129, ASP161, CYS163, GLY164)	None
OBP	3-Methylphenol	−5.5	8 (PHE35, VAL37, PHE55, VAL80, TYR82, ASN86, PHE88, ASN102)	None
	Lauric Acid	−4.2	9 (SER41, GLU43, PHE44, ASP45, TYR52, LEU53, ASN54, SER67, LEU68)	None
	Myristic Acid	−3.9	6 (ILE42, GLU43, ASP45, TYR52, LEU53, ASN54)	1 (SER41)
	Oleic Acid	−4.1	9 (LYS28, PRO34, GLU59, PHE66, TYR82, ALA83, ASP106, GLU107, GLU108)	1 (GLU27)
	Palmitic Acid	−4.3	10 (PHE10, GLU11, LEU12, PHE44, ASP46, LYS72, ASN76, TYR78, VAL90, ALA93)	None
	Skatole	−6.6	8 (PHE35, VAL37, PHE55, LEU68, TYR82, ASN86, ASN102, MET114)	None
	Tiglic aldehyde	−4.3	9 (THR96, ALA97, LEU117, GLY119, LYS120, ASP123, ILE124, GLU125, ASP128)	1 (GLY121)
Pheromaxein	3-Methylphenol	−4.7	7 (VAL94, LYS95, PHE98, PRO99, ILE101, LEU105, PHE110)	1 (SER102)
	Lauric Acid	−4.5	7 (GLU30, PHE31, PHE34, LEU35, LYS59, LEU77, LEU81)	None
	Myristic Acid	−4.9	7 (GLU30, PHE31, PHE34, LEU35, LYS59, VAL62, LEU81)	None
	Oleic Acid	−5.7	11 (PHE31, PHE34, LEU35, ALA55, ALA58, LYS59, VAL62, LEU77, THR80, LEU81, ILE84)	None
	Palmitic Acid	−5.3	8 (PHE31, PHE34, LEU35, VAL62, LYS59, LEU77, THR80, LEU81)	None
	Skatole	−4.8	4 (PHE31, PHE34, LEU35, LEU81)	1 (LEU77)
	Tiglic aldehyde	−3.7	7 (ALA26, LYS29, GLU30, ALA33, GLU45, LEU46, PHE49)	None
SAL	3-Methylphenol	−6.2	9 (VAL59, VAL61, PHE74, PHE76, CYS91, PHE109, LEU122, TYR139, GLU137)	None
	Lauric Acid	−3.4	8 (PHE29, ARG110, LEU111, LEU112, GLU113, ILE156, GLN159, TYR160)	1 (THR26)
	Myristic Acid	−4	10 (VAL98, THR100, ASN107, LYS108, PHE109, ARG110, HIS123, LEU124, VAL125, VAL127)	None
	Oleic Acid	−4.4	9 (LEU42, ASP144, PRO147, LYS150, ASP151, VAL154, LYS164, ILE167, LYS172)	2 (ASP169, THR171)
	Palmitic Acid	−4.2	11 (VAL98, THR100, GLU106, ASN107, LYS108, PHE109, ARG110, HIS123, LEU124, VAL125, VAL127)	None
	Skatole	−6.7	11 (VAL59, PHE74, PHE76, ALA89, CYS91, TYR103, VAL101, PHE109, LEU122, GLU137, TYR139)	None
	Tiglic aldehyde	−4.5	7 (PHE74, PHE76, ALA89, CYS91, VAL101, PHE109, TYR139)	None
VEGP	3-Methylphenol	−4.7	7 (MET41, LYS50, PHE74, ILE76, GLN81, VAL83, LYS133)	None
	Lauric Acid	−3.9	8 (ILE47, PHE74, ILE76, VAL83, TYR99, LEU124, MET131, LYS133)	2 (LYS50, GLN81)
	Myristic Acid	−3.7	8 (VAL25, GLY26, GLN27, LEU29, LYS89, PRO93, PHE94, PHE96)	None
	Oleic Acid	−3.8	10 (ALA24, VAL25, PRO28, LEU29, LYS89, THR90, GLN92, PRO93, PHE94, PHE96)	2 (GLY26, GLN27)
	Palmitic Acid	−4.7	11 (VAL25, GLY26, GLN27, LEU29, GLY66, LYS89, THR90, GLN92, PRO93, PHE94, PHE96)	1 (THR95)
	Skatole	−5.4	9 (LEU58, ALA70, LEU85, LEU87, VAL105, LEU118, CYS120, MET131, LYS133)	None
	Tiglic aldehyde	−4.1	5 (LEU38, LEU58, ILE72, PHE74, LEU118)	1 (LYS133)

### Molecular dynamics simulation

The stability of the orientation of the compounds with the receptor proteins can be confirmed in the dynamic environment using molecular dynamics simulations. The simulation was performed for 3-methylphenol (3METP), lauric acid (LAUA), myristic acid (MYRA), oleic acid (OLEA), palmitic acid (PALA), skatole (SKA), and tiglic aldehyde (TIGAL) - AGP, OBP, SAL, Pheromaxein, and VEGP complexes. For the MDS run lasting 100 ns, all 35 complexes were selected based on their binding energy and non-bonding interactions with the essential amino acid residues.

### RMSD and RMSF

**FIGURE 6:**
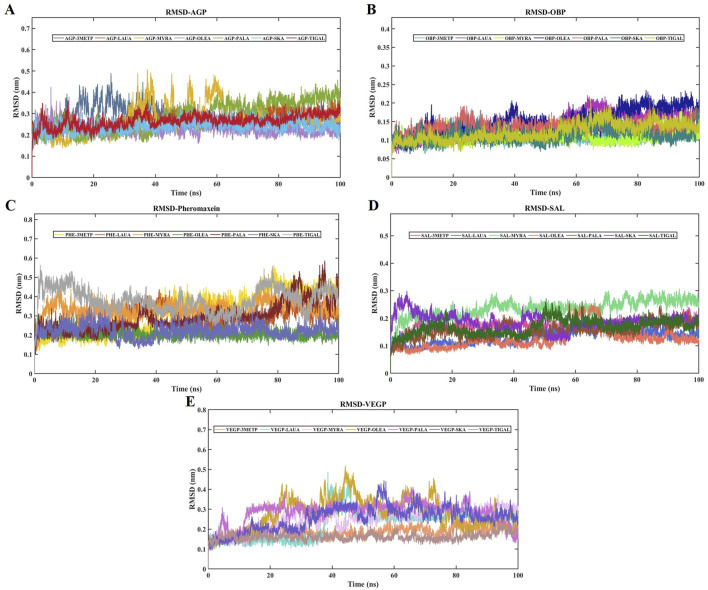
RMSDs of all the studied protein-ligand complexes for 100 ns each. **(A)** RMSD plot of AGP-3-Methylphenol (Blue-Gray), AGP-Lauric Acid (Burnt Orange), AGP-Myristic Acid (Mustard Yellow), AGP-Oleic Acid (Lavender), AGP-Palmitic Acid (Seafoam Green), AGP-Skatole (Sky Blue), and AGP-Tiglic aldehyde (Brick Red). **(B)** RMSD plot of OBP-3-Methylphenol (Turquoise), OBP-Lauric Acid (Deep Magenta), OBP-Myristic Acid (Lime), OBP-Oleic Acid (Navy Blue), OBP-Palmitic Acid (Salmon pink), OBP-Skatole (Teal Green), and OBP-Tiglic aldehyde (yellow-green) **(C)** RMSD plot of Pheromaxein-3-Methylphenol (Gold), Pheromaxein-Lauric Acid (Indigo), Pheromaxein-Myristic Acid (Tangerine), Pheromaxein-Oleic Acid (Forest Green), Pheromaxein-Palmitic Acid (Dark Red), Pheromaxein-Skatole (Pale Blue), and Pheromaxein-Tiglic aldehyde (Silver Gray) **(D)** RMSD plot of SAL-3-Methylphenol (Hot Pink), SAL-Lauric Acid (Royal Blue), SAL-Myristic Acid (Mint Green), SAL-Oleic Acid (Tomato), SAL-Palmitic Acid (Sienna Brown), SAL-Skatole (Violet), and SAL-Tiglic aldehyde (Deep green) **(E)** RMSD plot of VEGP-3-Methylphenol (Peach), VEGP-Lauric Acid (Aquamarine), VEGP-Myristic Acid (Lavender Blush), VEGP-Oleic Acid (Goldenrod), VEGP-Palmitic Acid (Orchid), VEGP-Skatole (Slate Blue), and VEGP-Tiglic aldehyde (Rosy Brown).

**FIGURE 7:**
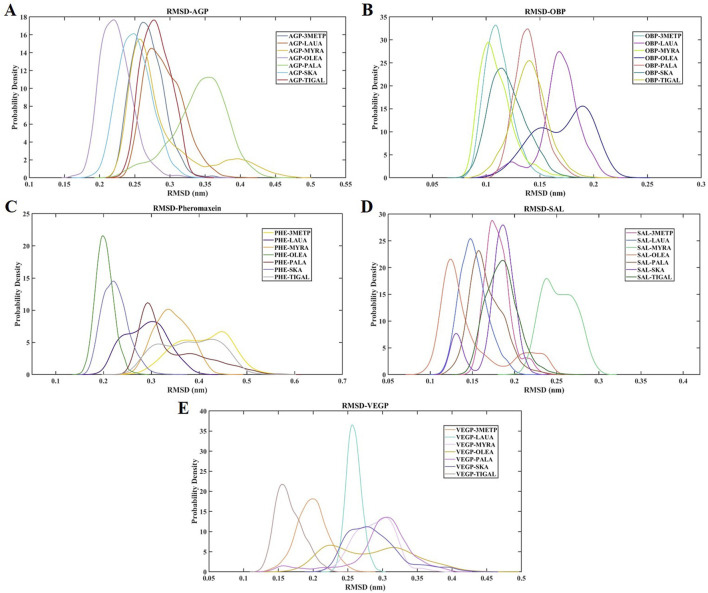
The RMSD probability distribution (50–100 ns) of all the studied protein-ligand complexes. **(A)** RMSD probability distribution plot of AGP-3-Methylphenol (Blue-Gray), AGP-Lauric Acid (Burnt Orange), AGP-Myristic Acid (Mustard Yellow), AGP-Oleic Acid (Lavender), AGP-Palmitic Acid (Seafoam Green), AGP-Skatole (Sky Blue), and AGP-Tiglic aldehyde (Brick Red). **(B)** RMSD probability distribution plot of OBP-3-Methylphenol (Turquoise), OBP-Lauric Acid (Deep Magenta), OBP-Myristic Acid (Lime), OBP-Oleic Acid (Navy Blue), OBP-Palmitic Acid (Salmon pink), OBP-Skatole (Teal Green), and OBP-Tiglic aldehyde (yellow-green) **(C)** RMSD probability distribution plot of Pheromaxein-3-Methylphenol (Gold), Pheromaxein-Lauric Acid (Indigo), Pheromaxein-Myristic Acid (Tangerine), Pheromaxein-Oleic Acid (Forest Green), Pheromaxein-Palmitic Acid (Dark Red), Pheromaxein-Skatole (Pale Blue), and Pheromaxein-Tiglic aldehyde (Silver Gray) **(D)** RMSD probability distribution plot of SAL-3-Methylphenol (Hot Pink), SAL-Lauric Acid (Royal Blue), SAL-Myristic Acid (Mint Green), SAL-Oleic Acid (Tomato), SAL-Palmitic Acid (Sienna Brown), SAL-Skatole (Violet), and SAL-Tiglic aldehyde (Deep green) **(E)** RMSD probability distribution plot of VEGP-3-Methylphenol (Peach), VEGP-Lauric Acid (Aquamarine), VEGP-Myristic Acid (Lavender Blush), VEGP-Oleic Acid (Goldenrod), VEGP-Palmitic Acid (Orchid), VEGP-Skatole (Slate Blue), and VEGP-Tiglic aldehyde (Rosy Brown).

**FIGURE 8:**
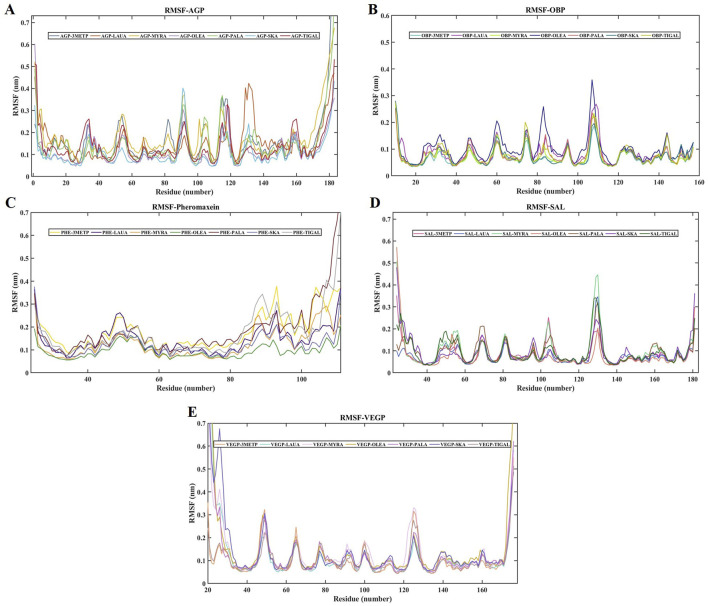
RMSF of all the studied protein-ligand complexes for 100 ns each. **(A)** RMSF plot of AGP-3-Methylphenol (Blue-Gray), AGP-Lauric Acid (Burnt Orange), AGP-Myristic Acid (Mustard Yellow), AGP-Oleic Acid (Lavender), AGP-Palmitic Acid (Seafoam Green), AGP-Skatole (Sky Blue), and AGP-Tiglic aldehyde (Brick Red). **(B)** RMSF plot of OBP-3-Methylphenol (Turquoise), OBP-Lauric Acid (Deep Magenta), OBP-Myristic Acid (Lime), OBP-Oleic Acid (Navy Blue), OBP-Palmitic Acid (Salmon pink), OBP-Skatole (Teal Green), and OBP-Tiglic aldehyde (yellow-green) **(C)** RMSF plot of Pheromaxein-3-Methylphenol (Gold), Pheromaxein-Lauric Acid (Indigo), Pheromaxein-Myristic Acid (Tangerine), Pheromaxein-Oleic Acid (Forest Green), Pheromaxein-Palmitic Acid (Dark Red), Pheromaxein-Skatole (Pale Blue), and Pheromaxein-Tiglic aldehyde (Silver Gray) **(D)** RMSF plot of SAL-3-Methylphenol (Hot Pink), SAL-Lauric Acid (Royal Blue), SAL-Myristic Acid (Mint Green), SAL-Oleic Acid (Tomato), SAL-Palmitic Acid (Sienna Brown), SAL-Skatole (Violet), and SAL-Tiglic aldehyde (Deep green) **(E)** RMSF plot of VEGP-3-Methylphenol (Peach), VEGP-Lauric Acid (Aquamarine), VEGP-Myristic Acid (Lavender Blush), VEGP-Oleic Acid (Goldenrod), VEGP-Palmitic Acid (Orchid), VEGP-Skatole (Slate Blue), and VEGP-Tiglic aldehyde (Rosy Brown).

The RMSD of the protein relative to the protein backbone was calculated and depicted in Figure 6. The stability of the complex (protein-ligand) increases as the RMSD decreases. RMSDs of all protein-ligand systems revealed minimal variation from their average structure, ranging from 0.11 ± 0.01 nm to 0.38 ± 0.06 nm (Table 2). For the final 50 ns of the simulation, all protein complexes reached equilibrium, as indicated by a smooth curve, despite a few fluctuations in the MDS trajectory. Stability was determined using statistical validation, with the probability distribution of backbone RMSDs generated for all simulations ranging from 50 to 100 ns (Figure 7). The RMSF was estimated from the MDS trajectory for the Cα atom of the protein and was found to be below 0.2 nm in all protein complexes, indicating that the complexes are stable. Figure 8 shows the RMSF profile, illustrating the variation in the Cα atoms within the amino acid residues. The spikes in the amino acid residue RMSF graphs may be attributed to the unstable, fluctuating loop structures. The average and standard deviations of the RMSF are listed in Table 2.

**TABLE 2:** Average and standard deviation values for RMSD, RMSF, and maximum number of H-bonds formed in the molecular dynamics simulation of the complexes.

Complexes	RMSD	RMSF	Maximum H-bond
AGP-3-Methylphenol	0.28 ± 0.04	0.16 ± 0.12	2
AGP-Lauric Acid	0.28 ± 0.03	0.15 ± 0.08	6
AGP-Myristic Acid	0.27 ± 0.07	0.18 ± 0.10	4
AGP-Oleic Acid	0.23 ± 0.03	0.12 ± 0.07	5
AGP-Palmitic Acid	0.29 ± 0.07	0.15 ± 0.10	5
AGP-Skatole	0.24 ± 0.03	0.11 ± 0.07	2
AGP-Tiglic aldehyde	0.27 ± 0.03	0.14 ± 0.08	2
OBP-3-Methylphenol	0.11 ± 0.02	0.07 ± 0.03	2
OBP-Lauric Acid	0.14 ± 0.03	0.09 ± 0.04	4
OBP-Myristic Acid	0.11 ± 0.01	0.07 ± 0.04	4
OBP-Oleic Acid	0.15 ± 0.03	0.10 ± 0.05	2
OBP-Palmitic Acid	0.13 ± 0.02	0.08 ± 0.04	4
OBP-Skatole	0.11 ± 0.02	0.07 ± 0.04	2
OBP-Tiglic aldehyde	0.12 ± 0.02	0.08 ± 0.04	2
Pheromaxein-3-Methylphenol	0.32 ± 0.11	0.18 ± 0.08	3
Pheromaxein-Lauric Acid	0.28 ± 0.06	0.14 ± 0.06	3
Pheromaxein-Myristic Acid	0.33 ± 0.04	0.13 ± 0.06	1
Pheromaxein-Oleic Acid	0.20 ± 0.02	0.10 ± 0.03	2
Pheromaxein-Palmitic Acid	0.29 ± 0.07	0.19 ± 0.12	1
Pheromaxein-Skatole	0.22 ± 0.04	0.13 ± 0.05	2
Pheromaxein-Tiglic aldehyde	0.38 ± 0.06	0.18 ± 0.10	1
SAL-3-Methylphenol	0.17 ± 0.02	0.08 ± 0.05	3
SAL-Lauric Acid	0.13 ± 0.03	0.08 ± 0.05	3
SAL-Myristic Acid	0.23 ± 0.03	0.10 ± 0.08	3
SAL-Oleic Acid	0.13 ± 0.04	0.08 ± 0.06	4
SAL-Palmitic Acid	0.15 ± 0.02	0.09 ± 0.05	4
SAL-Skatole	0.19 ± 0.03	0.09 ± 0.06	2
SAL-Tiglic aldehyde	0.17 ± 0.03	0.09 ± 0.06	1
VEGP-3-Methylphenol	0.19 ± 0.03	0.10 ± 0.07	3
VEGP-Lauric Acid	0.22 ± 0.07	0.12 ± 0.13	4
VEGP-Myristic Acid	0.25 ± 0.05	0.13 ± 0.11	3
VEGP-Oleic Acid	0.28 ± 0.08	0.14 ± 0.15	3
VEGP-Palmitic Acid	0.28 ± 0.05	0.13 ± 0.12	5
VEGP-Skatole	0.25 ± 0.06	0.14 ± 0.13	2
VEGP-Tiglic aldehyde	0.16 ± 0.02	0.10 ± 0.06	2

### Intermolecular hydrogen bond analysis

We observed the maximum number of intermolecular hydrogen bonds (H-bonds) among the complexes over 100 ns in the MDS (Table 2), and the stability of the complexes increased with the number of hydrogen bonds. Specifically, AGP-lauric acid, AGP-oleic acid, AGP-palmitic acid, AGP-myristic acid, OBP-lauric acid, OBP-myristic acid, OBP-palmitic acid, SAL-oleic acid, SAL-palmitic acid, VEGP-lauric acid, and VEGP-palmitic acid complexes showed the highest number of hydrogen bonds (four to six) at various time points during MDS (Supplementary Figures S2-6).

### MM/PBSA calculation

The binding free energy change was used to assess the spontaneity and feasibility of complex formation, as shown in Table 3. The smaller the values of the change in binding free energy, the better thermodynamically stable the complex; a value less than zero indicates that the response is spontaneous. The predicted binding free energy changes for the AGP-3-methylphenol, AGP-lauric acid, AGP-myristic acid, AGP-oleic acid, and AGP-palmitic acid complexes were −65.573 ± 7.555, −157.312 ± 11.141, −161.364 ± 15.386, −153.944 ± 16.705, and −177.532 ± 15.776 kJ/mol, respectively. Unexpectedly, the predicted binding free energy changes for AGP-skatole were positive, while AGP-tiglic aldehyde was −8.001 ± 38.682 kJ/mol (Table 3; Supplementary Figure S7). Among the OBP complexes, OBP-skatole showed the more negative binding energy (−83.004 ± 5.463 kJ/mol). OBP-3-methylphenol, OBP-myristic acid, and OBP-palmitic acid showed almost similar binding energies (−80.378 ± 6.292, −68.021 ± 19.111, and −77.451 ± 20.772 kJ/mol, respectively). In contrast, OBP-lauric acid (−20.173 ± 36.388 kJ/mol), OBP-oleic acid (−57.490 ± 34.420 kJ/mol), and OBP-tiglic aldehyde (−27.613 ± 26.043 kJ/mol) had the least similar binding energies in the OBP complexes (Table 3; Supplementary Figure S7). The binding free energies for the pheromaxein complexes exhibited a significant variation; the more negative binding energies were for pheromaxein-lauric acid, pheromaxein-myristic acid, pheromaxein-oleic acid, and pheromaxein-palamitic acid (−93.500 ± 8.826, −105.631 ± 12.652, −107.693 ± 13.774, and −114.967 ± 14.125 kJ/mol, respectively), whereas the less negative for pheromaxein-3-methylphenol, pheromaxein-skatole, and pheromaxein-tiglic aldehyde (−22.700 ± 25.261, −20.420 ± 30.812, and −23.563 ± 26.677 kJ/mol, respectively) (Table 3; Supplementary Figure S7). Similarly, SAL complexes also showed a significant variation in binding energy; the more negative binding energies were found in SAL-3-methylphenol (−70.448 ± 9.275), SAL-lauric acid (−69.813 ± 11.775), SAL-myristic acid (−86.449 ± 12.085), SAL-palmitic acid (−77.193 ± 35.090), and SAL-skatole (−76.113 ± 8.814). The slightly less negative binding energies were found with SAL-oleic acid (−51.935 ± 19.799) and SAL-tiglic aldehyde (−51.237 ± 8.261) (Table 3; Supplementary Figure S7). The binding free energies for the VEGP complexes were more negative for VEGP-lauric (−118.807 ± 24.114 kJ/mol), VEGP-myristic (−144.099 ± 11.605 kJ/mol), VEGP-oleic (−167.288 ± 15.479 kJ/mol), and VEGP-palmitic acid (−69.120 ± 24.771 kJ/mol), while less negative for VEGP-3-methylphenol (−48.541 ± 20.753 kJ/mol), VEGP-skatole (−23.293 ± 33.044 kJ/mol), and VEGP-tiglic aldehyde (−51.025 ± 9.100 kJ/mol) (Table 3; Supplementary Figure S7). Overall, most of the high free binding energies were observed for fatty acids when bound to different proteins.

**TABLE 3:** MM/PBSA calculation of various complexes, including Van der Waals energy, electrostatic energy, SASA energy, and total binding energy.

Complexes	Van der Waals energy ( ± SD) (kJ/mol)	Electrostatic energy ( ± SD) (kJ/mol)	Polar solvation energy ( ± SD) (kJ/mol)	SASA energy ( ± SD) (kJ/mol)	Total binding energy ( ± SD) (kJ/mol)
AGP-3-Methylphenol	−70.641 ± 6.653	−14.495 ± 4.323	29.174 ± 4.155	−9.611 ± 0.605	−65.573 ± 7.555
AGP-Lauric Acid	−105.117 ± 11.832	−102.197 ± 10.421	65.139 ± 5.916	−15.137 ± 0.666	−157.312 ± 11.141
AGP-Myristic Acid	−142.324 ± 11.903	−70.958 ± 25.255	70.597 ± 14.757	−18.679 ± 0.734	−161.364 ± 15.386
AGP-Oleic Acid	−170.604 ± 10.565	−10.577 ± 16.214	46.732 ± 12.385	−19.495 ± 0.886	−153.944 ± 16.705
AGP-Palmitic Acid	−151.725 ± 11.877	−98.809 ± 14.172	93.388 ± 17.180	−20.387 ± 0.801	−177.532 ± 15.776
AGP-Skatole	−3.207 ± 6.807	−0.021 ± 1.617	6.890 ± 43.603	−0.714 ± 1.782	2.947 ± 43.748
AGP-Tiglic aldehyde	−9.676 ± 13.661	−1.715 ± 3.922	5.010 ± 35.984	−1.620 ± 2.693	−8.001 ± 38.682
OBP-3-Methylphenol	−65.524 ± 7.811	−21.080 ± 3.837	15.284 ± 1.493	−9.058 ± 0.524	−80.378 ± 6.292
OBP-Lauric Acid	−20.657 ± 25.363	−8.176 ± 15.234	12.431 ± 37.978	−3.770 ± 3.589	−20.173 ± 36.388
OBP-Myristic Acid	−66.664 ± 13.991	−61.545 ± 31.620	71.895 ± 27.150	−11.707 ± 1.696	−68.021 ± 19.111
OBP-Oleic Acid	−64.019 ± 29.892	−29.796 ± 18.060	46.604 ± 28.279	−10.278 ± 3.845	−57.490 ± 34.420
OBP-Palmitic Acid	−88.733 ± 14.773	−40.030 ± 19.367	65.498 ± 18.879	−14.186 ± 1.715	−77.451 ± 20.772
OBP-Skatole	−71.839 ± 5.906	−12.911 ± 5.909	11.698 ± 2.537	−9.952 ± 0.593	−83.004 ± 5.463
OBP-Tiglic aldehyde	−25.405 ± 20.465	−6.070 ± 10.166	7.697 ± 20.122	−3.836 ± 2.848	−27.613 ± 26.043
Pheromaxein-3-Methylphenol	−37.380 ± 22.509	−5.972 ± 12.818	26.418 ± 21.667	−5.767 ± 2.988	−22.700 ± 25.261
Pheromaxein-Lauric Acid	−92.907 ± 9.133	−1.673 ± 5.031	13.414 ± 7.974	−12.334 ± 0.881	−93.500 ± 8.826
Pheromaxein-Myristic Acid	−101.401 ± 11.187	−0.514 ± 2.783	9.694 ± 3.396	−13.410 ± 0.971	−105.631 ± 12.652
Pheromaxein-Oleic Acid	−105.522 ± 11.445	−1.640 ± 4.523	14.323 ± 9.219	−14.855 ± 1.340	−107.693 ± 13.774
Pheromaxein-Palmitic Acid	−113.695 ± 12.620	−4.661 ± 9.630	18.645 ± 14.655	−15.256 ± 1.733	−114.967 ± 14.125
Pheromaxein-Skatole	−25.036 ± 17.357	−3.207 ± 5.707	12.290 ± 27.903	−4.467 ± 2.867	−20.420 ± 30.812
Pheromaxein-Tiglic aldehyde	−23.774 ± 17.596	−1.626 ± 6.593	5.917 ± 19.711	−4.080 ± 2.874	−23.563 ± 26.677
SAL-3-Methylphenol	−59.279 ± 10.325	−34.583 ± 12.959	32.317 ± 6.270	−8.902 ± 0.661	−70.448 ± 9.275
SAL-Lauric Acid	−62.730 ± 9.196	−36.630 ± 7.616	39.694 ± 8.219	−10.147 ± 0.873	−69.813 ± 11.775
SAL-Myristic Acid	−110.133 ± 11.600	−15.006 ± 12.977	53.632 ± 11.690	−14.943 ± 1.357	−86.449 ± 12.085
SAL-Oleic Acid	−57.809 ± 15.224	−16.054 ± 21.568	31.590 ± 30.735	−9.662 ± 1.841	−51.935 ± 19.799
SAL-Palmitic Acid	−77.411 ± 30.314	−65.563 ± 34.578	78.704 ± 38.341	−12.923 ± 3.954	−77.193 ± 35.090
SAL-Skatole	−76.008 ± 5.314	−20.338 ± 13.206	30.816 ± 5.482	−10.583 ± 0.590	−76.113 ± 8.814
SAL-Tiglic aldehyde	−56.623 ± 4.183	−5.031 ± 7.066	18.689 ± 5.009	−8.273 ± 0.524	−51.237 ± 8.261
VEGP-3-Methylphenol	−47.526 ± 21.027	−20.551 ± 14.488	26.421 ± 23.573	−6.886 ± 2.846	−48.541 ± 20.753
VEGP-Lauric Acid	−124.381 ± 23.490	−22.684 ± 14.142	44.217 ± 14.893	−15.959 ± 2.402	−118.807 ± 24.114
VEGP-Myristic Acid	−149.162 ± 9.821	−7.271 ± 8.536	31.528 ± 5.488	−19.194 ± 0.944	−144.099 ± 11.605
VEGP-Oleic Acid	−168.476 ± 12.877	−19.789 ± 12.577	42.860 ± 10.611	−21.883 ± 1.241	−167.288 ± 15.479
VEGP-Palmitic Acid	−75.874 ± 16.475	−14.553 ± 17.905	32.311 ± 14.664	−11.003 ± 2.231	−69.120 ± 24.771
VEGP-Skatole	−23.067 ± 22.347	−14.677 ± 18.404	18.251 ± 36.886	−3.799 ± 3.251	−23.293 ± 33.044
VEGP-Tiglic aldehyde	−50.324 ± 8.493	−17.029 ± 15.163	24.631 ± 10.644	−8.303 ± 0.615	−51.025 ± 9.100

Furthermore, the receptor protein–ligand complexes binding free energy (ΔG) was determined by integrating configurational entropy changes with MM/PBSA techniques. Using the thermodynamic relationship, the total binding free energy was calculated (ΔG = ΔH–TΔS). The enthalpic component, represented by ΔH, was obtained using MM/PBSA calculations that included contributions from van der Waals, electrostatic, polar solvation, and non-polar solvation. When estimating the entropic component TΔS, the mass-weighted covariance matrix of atomic fluctuations taken from equilibrated MDS trajectories was subjected to the Quasi-Harmonic Approximation. T = 300 K was used to convert the entropy output (in J/mol K) to energy units (kJ/mol), which were then subtracted from ΔH to obtain the final ΔG. Supplementary Table S4 shows the obtained binding free energy, as well as the change in enthalpy and entropy for all complexes. Conformational entropy losses during complex formation for all complexes are accounted for, improving the precision of binding affinity estimations.

## Discussion

Odorant-binding proteins play a crucial role in mammals by receiving and processing chemical cues, and then transferring neuronal signals to the brain, ultimately leading to neuroendocrine action. The quality and quantity of each chemical present in the cue determine the intensity and extent of action. Although behavioral studies provide real-time evidence for a particular chemical cue, the efficacy of binding of the cues to the protein can be assessed either through *in vivo* or *in silico* approaches. In this context, computational tools offer valuable insights into evaluating the efficacy of ligand binding to proteins. In this study, we aimed to assess the binding efficacy of compounds derived from pig secretions with various receptor proteins. It is essential to test these compounds, as they have been reported to help mitigate the weaning stress in piglets. The docked complexes (compounds bound to the protein) exhibited variations in the docking scores. The more negative the docking score, the higher the binding efficiency. We found the less negative docking score for pheromaxein and tiglic aldehyede (−3.7 kcal/mol) and the more negative score for SAL and skatole (−6.7 kcal/mol). AGP showed an average score of −5 kcal/mol for all compounds except tiglic aldehyde. The scores of the other complexes (OBP-ligands, pheromaxein-ligands, SAL-ligands, and VEGP-ligands) were highly variable. However, a few complexes exhibited hydrogen bonding interactions, indicating stability (Rajagopalan Vaidyanathan et al., 2023). According to the lowest RMSD and hydrogen bond formation in the pre-MDS analysis, molecular docking identified the best-docked complexes. These complexes include, myristic acid (−5.4 kcal/mol, hydrogen bonding with LYS88 and 14 other interacting residues) against AGP; myristic acid (−3.9 kcal/mol, hydrogen bonding with SER41 and 6 other interacting residues), oleic acid (−4.1 kcal/mol, hydrogen bonding with GLU27 and 9 other interacting residues), and tiglic aldehyde (−4.3 kcal/mol, hydrogen bonding with GLY121 and 9 other interacting residues) against OBP; 3 methylphenol (−4.7 kcal/mol, hydrogen bonding with SER102 and 7 other interacting residues), and skatole (−4.8 kcal/mol, hydrogen bonding with LEU77 and 4 other interacting residues) against pheromaxein; lauric acid (−3.4 kcal/mol, hydrogen bonding with THR26 and 8 other interacting residues), oleic acid (−4.4 kcal/mol, hydrogen bonding with ASP169 and THR171, and 9 other interacting residues) against SAL; and lauric acid (−3.9 kcal/mol, hydrogen bonding with LYS50 and GLN81 and 8 other interacting residues), oleic acid (−3.8 kcal/mol, hydrogen bonding with GLY26 and GLN27 and 10 other interacting residues), palmitic acid (−4.7 kcal/mol, hydrogen bonding with THR95 and 11 other interacting residues), and tiglic aldehyde (−4.1 kcal/mol, hydrogen bonding with LYS133 and 5 other interacting residues) against VEGP. They were superior to the others in terms of more significant interaction and binding affinities.

The docked complexes with fatty acids yielded more interacting hydrogen bonds, implying that these fatty acids likely contributed to the behavioral effects in piglets. A mixture of various fatty acids (including oleic acid) exhibited c-fos expression across multiple brain areas, such as the BNST, amygdala, and hypothalamus, which are highly important for coordinating neuroendocrine action (Li et al., 2022). Indeed, oleic acid and p-cresol exhibited high binding energy with buffalo nasal OBP (bunOBP) and have been proposed to transduce olfactory signaling as combined molecules (Muthukumar et al., 2018). Despite the good binding energy with all proteins, 3-methyl phenol (m-cresol) showed hydrogen bonding only with pheromaxein in the present study. In support of this, Karthikeyan et al. (2014) also demonstrated that p-cresol exhibited a strong interaction only with OBP, but not with *β*-lactoglobulin. It is proposed that skatole and myristic acid are secreted in the udder regions of sows, in that skatole is perceived by the piglets at long distances and that myristic acid is perceived when the piglets are closer to the source (McGlone et al., 2022); thus, the binding score of the molecules varied with different proteins in the present study.

MDS is a crucial computational method for studying dynamic interactions in ligand-protein complexes, offering benefits over molecular docking. Unlike traditional molecular docking, it addresses protein rigidity and enables ligand conformational changes within the active site of the protein-ligand complex. The fundamental rationale for using MDS is its ability to precisely mimic real biological scenarios. In MDS, RMSD and RMSF are two crucial parameters for assessing the stability of a protein in the presence of ligands. In the present study, we found highly stable complexes of OBP and SAL compared with other proteins, as evidenced by the RMSD values. In particular, RMSD values were high for the pheromaxein complex, which is consistent with our previous study on pheromaxein and sex pheromones in pigs (Sankarganesh et al., 2024). Although a high variation was observed in the RMSD of all complexes in the present study, the complexes were found to be stable, indicating a high likelihood of firm binding between the ligands and the proteins. The RMSD values for codlemone and (Z)-8-dodecenol in complex with pheromone-binding proteins of *Grapholita molesta* were also documented, confirming the binding efficacy of the complexes (Liu et al., 2022).

RMSF is also a critical parameter for assessing the stability of complexes; therefore, we examined the RMSF of the complexes and found high values for AGP, pheromaxein, and VEGP. In contrast, the OBP and SAL complexes exhibited lower RMSF values, indicating stable conformations. All fatty acids showed similar RMSF values with VEGP, whereas the values fluctuated for pheromaxein and fatty acids. VEGP showed apparent favoritism only for fatty acids but not for 3-methylphenol, skatole, and tiglic aldehyde, which implies that VEGP is one of the vital proteins involved in porcine chemical communication. The RMSF values of fatty acids with any of the proteins used in this study indicate that all the compounds variably interact with different proteins, but, on the whole, may function efficiently as a mixture. Indeed, a mixture of compounds was proposed to have synergistic effects in inducing behavioral and neuroendocrine changes (Wyatt, 2010).

The number of intermolecular hydrogen bonds between the protein and ligand molecule influences the stability of the complex. The change in the hydrogen bond count over the MDS determines the stability of the complexes. In the present study, the number of intermolecular hydrogen bonds, ranging from at least one to a maximum of six, indicates the overall stability of the complexes. In particular, all the fatty acids (palmitic acid, lauric acid, myristic acid, and oleic acid) exhibited a maximum number of hydrogen bonds with most proteins, indicating the highest possibility of acting as a molecule of interest. In contrast, tiglic aldehyde showed the least number of hydrogen bonds with most proteins, implying that it is less likely to be involved in porcine chemical communication. It is crucial to note that tiglic aldehyde has been identified as a rabbit maternal neonatal pheromone (Schaal et al., 2003) but has been proposed as an interomone for pigs (McGlone et al., 2019). Considering the species-specific behavioral effects of pheromones, tiglic aldehyde induced the highest level of response in rabbits. However, it is also possible that when provided in a mixture, the interomone may also produce a synergistic effect in combination with other molecules. It is demonstrated that the intermolecular hydrogen bond formation favors the binding of the ligand to the protein (specifically within the binding pocket) with high efficiency. The bond formation throughout the entire simulation period indicates the good stability of the complexes (Nagare et al., 2023). We found continuous intermolecular hydrogen bonds between all proteins (except pheromaxein) and all fatty acids, which attests to the stability of the complexes.

The absolute binding energy between the ligand and protein can be calculated using MM/PBSA (Genheden and Ryde, 2015), a highly reliable method (Zhu et al., 2022). When the entropic contribution (−TΔS) was included using the Quasi-Harmonic approximation, the absolute binding free energy (ΔG) values changed, but the relative increase in binding affinities among the protein–ligand complexes remains the same. This suggests that while entropy improves the thermodynamic precision of individual estimations, it has no apparent effect on the study’s comparative ranking of ligand binding strengths. In our study, lauric acid, myristic acid, palmitic acid, and oleic acid showed more negative scores for three to two proteins each, whereas skatole showed high scores for OBP and SAL. This suggests that fatty acids are promising molecules. Human serum albumin binds to two fatty acids (myristate and palmitate) at high-affinity (more negative energy) binding sites (Fujiwara and Amisaki, 2008). Some of the proteins used in the present study also share similar structural characteristics with serum albumin in that OBP is a lipocalin with a binding cavity for ligands. These binding pockets may exhibit more negative binding energy (high affinity) for fatty acids; therefore, fatty acids are preferably bound in them. Fatty acids may occupy the high-energy binding sites in albumin via a sequential mechanism (Rizzuti et al., 2015; Bello, 2014 highlighted that the large ligand size of the fatty acids may favor a stronger binding affinity (more negative binding energy) with β-lactoglobulin, as indicated by MM-PBSA energies. Accordingly, we propose that fatty acids may be highly favored for binding with different proteins. In the present study, skatole exhibited positive or less negative binding free energies with AGP and pheromaxein, indicating that the heterocyclic nature of skatole did not facilitate efficient binding with proteins.

However, the binding energies were more negative than those of the three proteins. In our previous study, the binding energies of quinoline with different proteins were lower affinity (higher binding energy) because of its heterocyclic nature (Sankarganesh et al., 2024). 3-methylphenol exhibited more negative binding free energies only with OBP and SAL, which were comparable to or less negative than the binding energies of fatty acids. Conversely, 3-methylphenol showed less negative energy with other proteins, and these values were similar to those of skatole and tiglic aldehyde. The fundamental issue of binding 3-methylphenol, skatole, and tiglic aldehyde could be due to their lower molecular weights compared to fatty acids. Therefore, we may expect that fatty acids have more negative binding free energies (higher binding affinities) than other compounds. It is also possible that some compounds present in the mixture may be attributed to a specific smell but may not elicit changes in the olfactory system. Therefore, it is reasonable to believe that fatty acids are highly promising molecules for the suppression of unwanted behaviors in weaned piglets.

## Data Availability

The datasets presented in this study can be found in online repositories. The names of the repository/repositories and accession number(s) can be found in the article/Supplementary Material.
