# Supplementary material for: In-silico evaluation of putative maternal semiochemicals of pigs with receptor proteins
Source: Front Mol Biosci. 2025 Aug 8;12:1600209. doi: 10.3389/fmolb.2025.1600209 (PMC12370525; doi:10.3389/fmolb.2025.1600209)
Supplement: Supplementary file 1 [file Supplementaryfile1.docx]

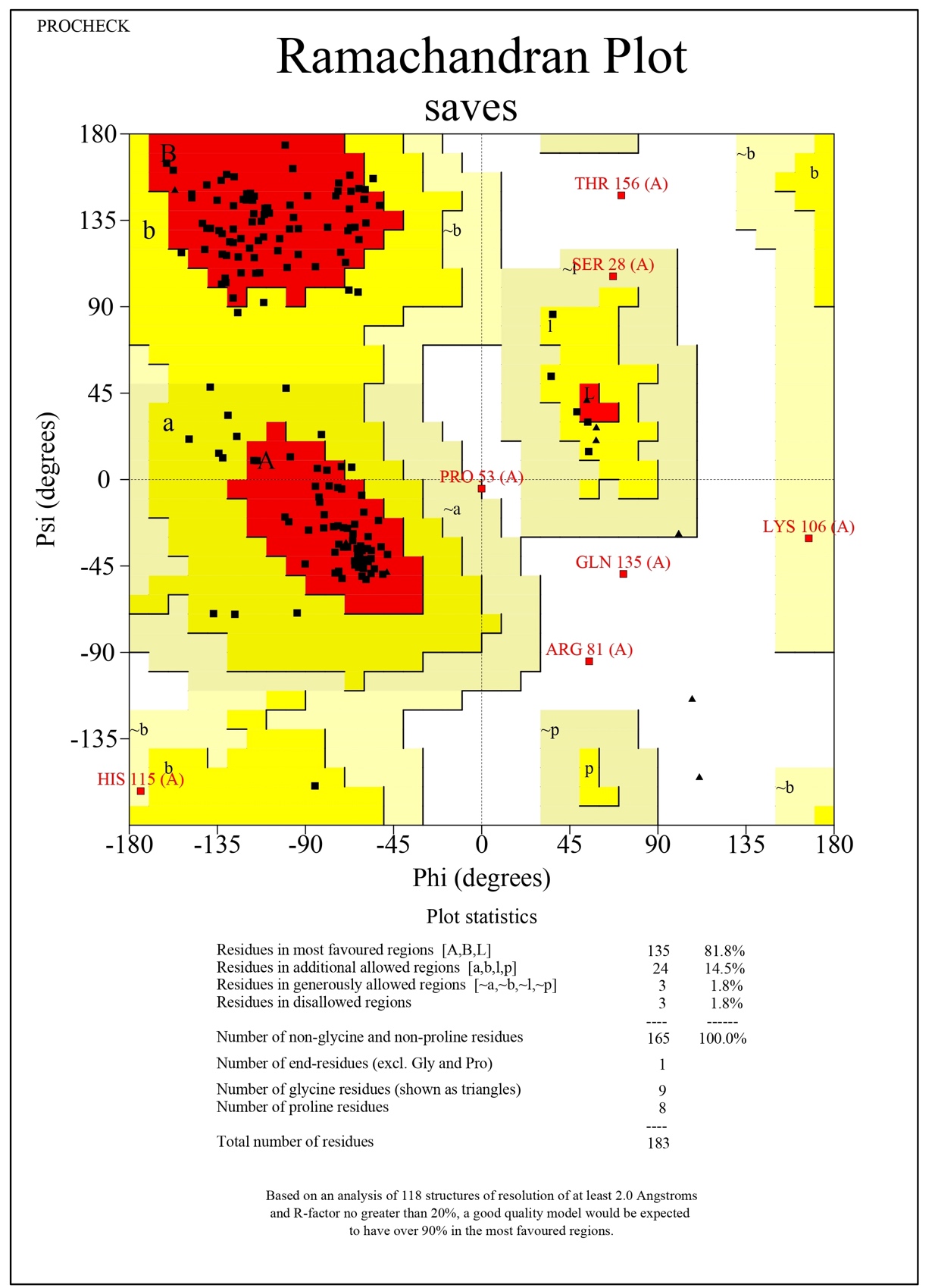


**Supplementary Figure 1:** Ramachandran plot for structural validation of modeled Alpha-1-acid glycoprotein using SAVES-PROCHECK.


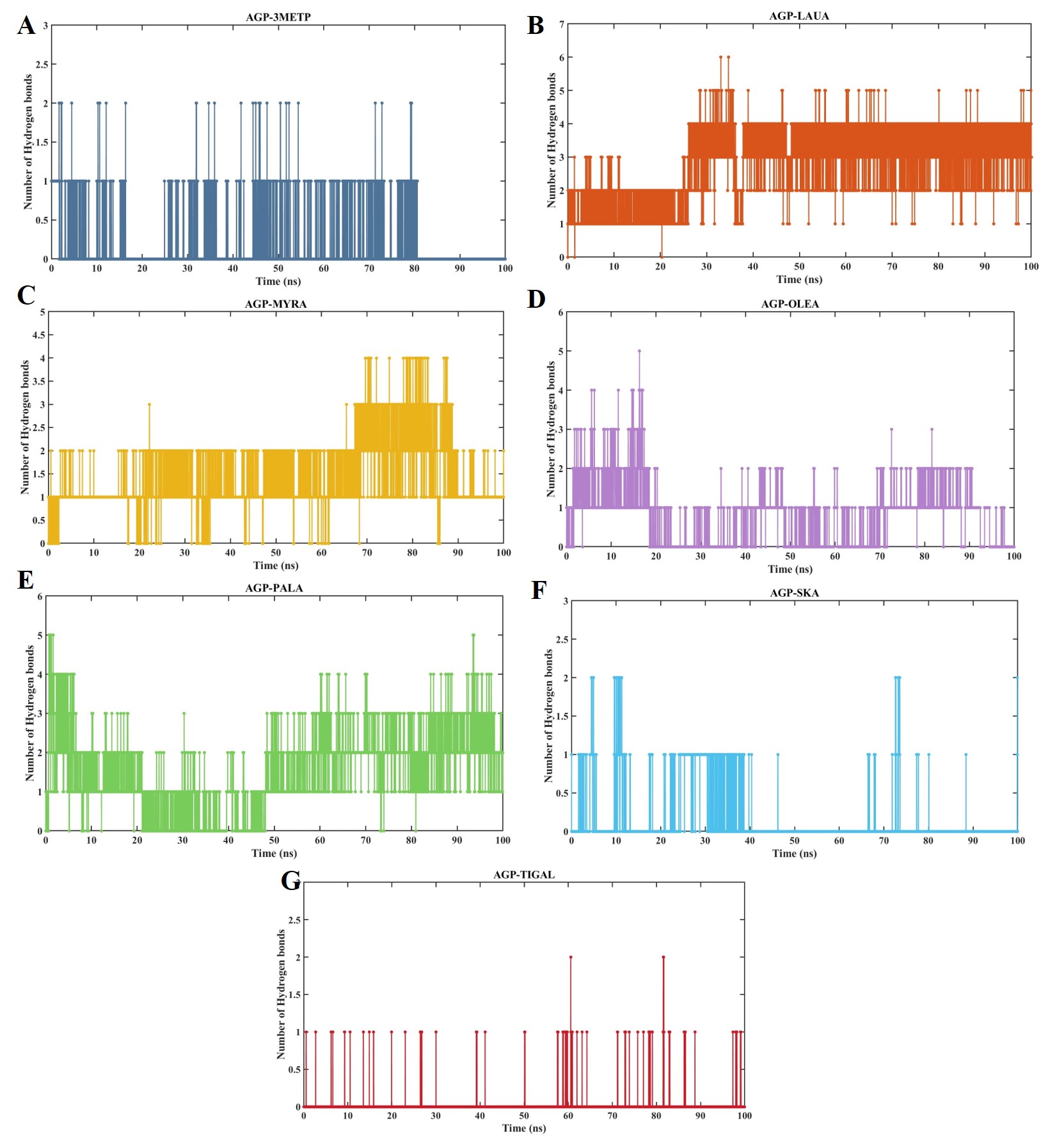


**Supplementary Figure 2:** Number of Intermolecular hydrogen bonds between compounds [3-Methylphenol (Blue-Gray), Lauric Acid (Burnt Orange), Myristic Acid (Mustard Yellow), Oleic Acid (Lavender), Palmitic Acid (Seafoam Green), Skatole (Sky Blue), and Tiglic aldehyde (Brick Red)] and AGP from MDS trajectory.


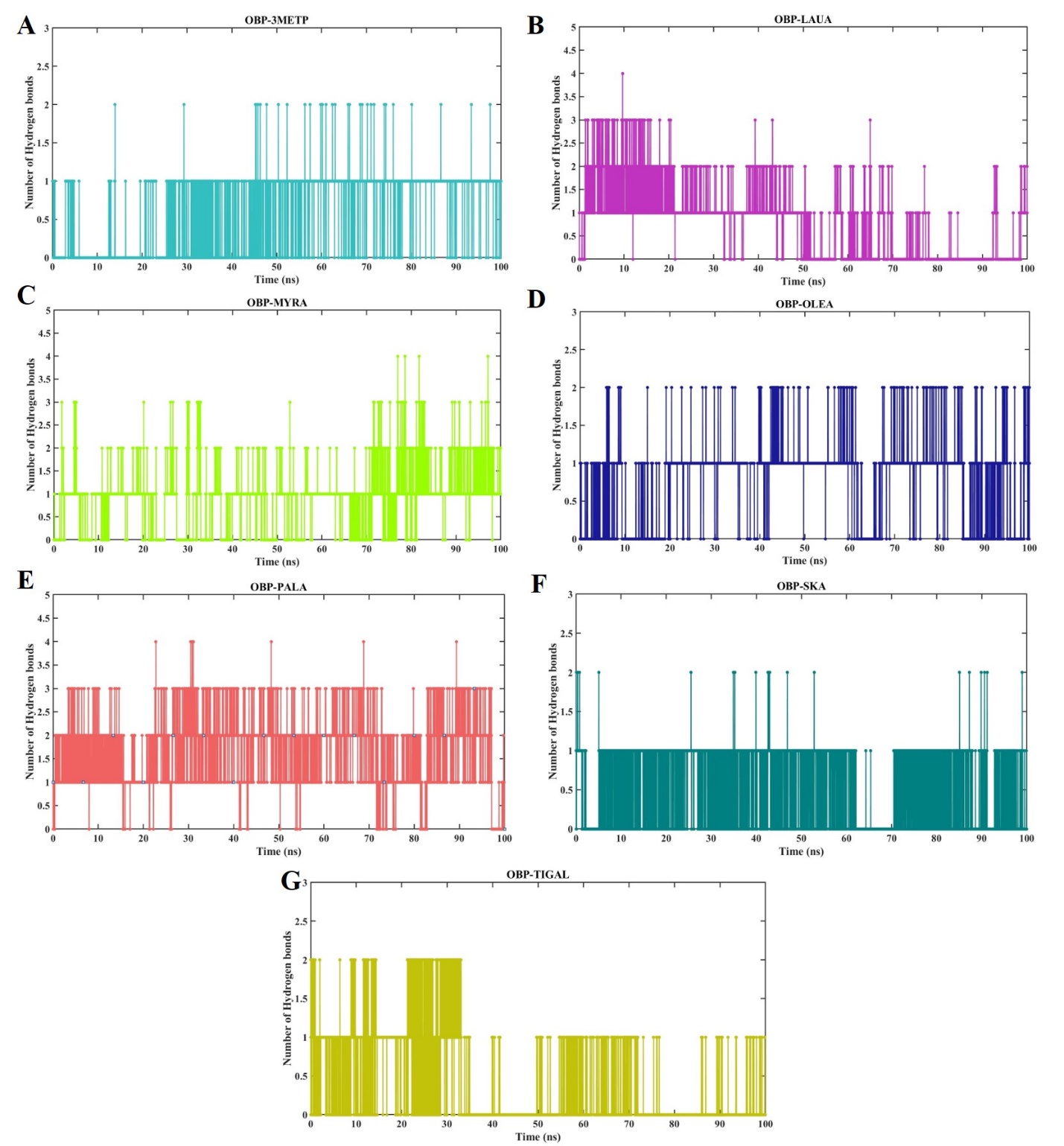


**Supplementary Figure 3:** Number of Intermolecular hydrogen bonds between compounds [3-Methylphenol (Turquoise), Lauric Acid (Deep Magenta), Myristic Acid (Lime), Oleic Acid (Navy Blue), Palmitic Acid (Salmon pink), Skatole (Teal Green), and Tiglic aldehyde (yellow-green)] and OBP from MDS trajectory.


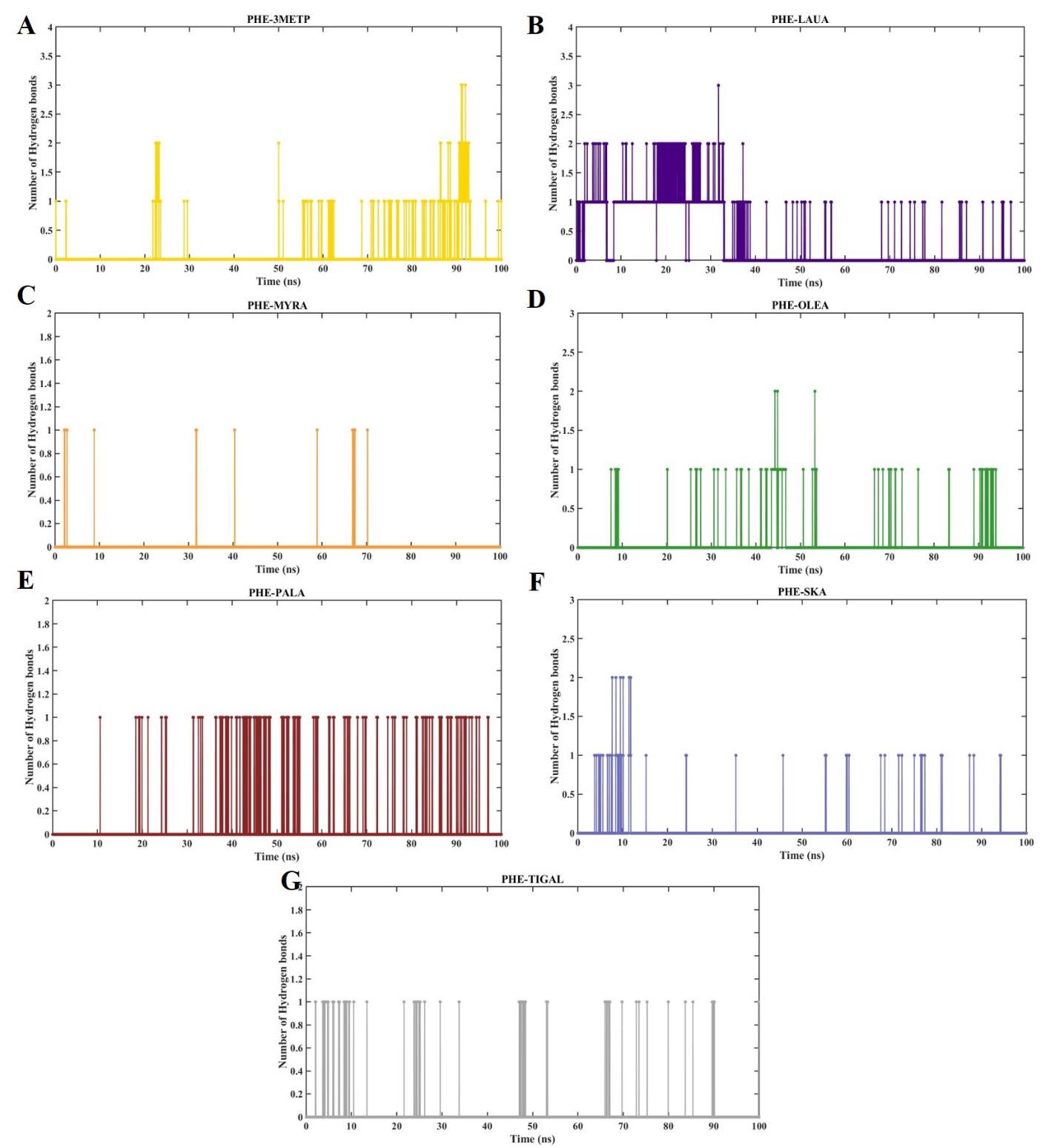


**Supplementary Figure 4:** Number of Intermolecular hydrogen bonds between compounds [3-Methylphenol (Gold), Lauric Acid (Indigo), Myristic Acid (Tangerine), Oleic Acid (Forest Green), Palmitic Acid (Dark Red), Skatole (Pale Blue), and Tiglic aldehyde (Silver Gray)] and Pheromaxein from MDS trajectory.


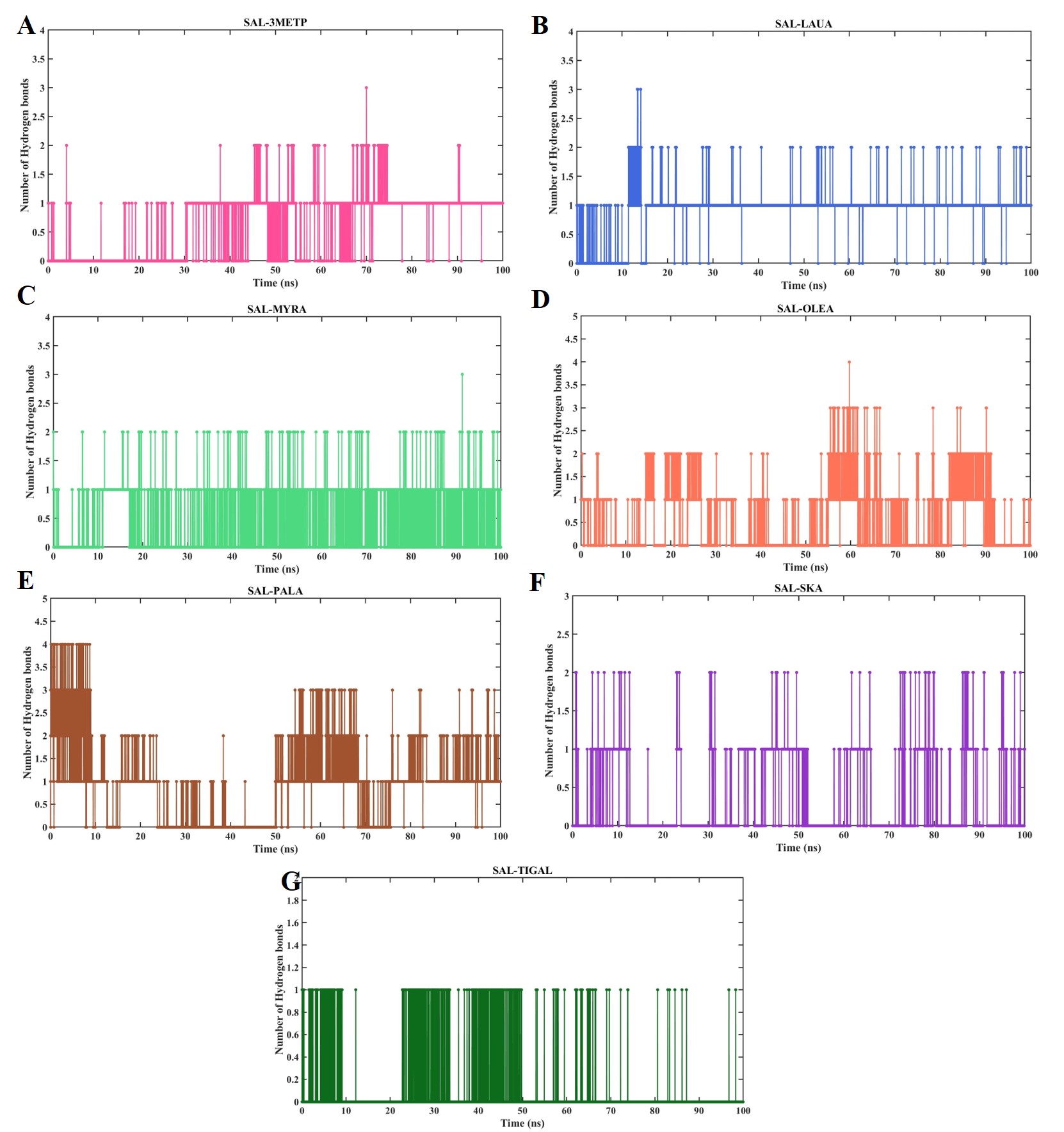


**Supplementary Figure 5:** Number of Intermolecular hydrogen bonds between compounds [3-Methylphenol (Hot Pink), Lauric Acid (Royal Blue), Myristic Acid (Mint Green), Oleic Acid (Tomato), Palmitic Acid (Sienna Brown), Skatole (Violet), and Tiglic aldehyde (Deep green)] and SAL from MDS trajectory.


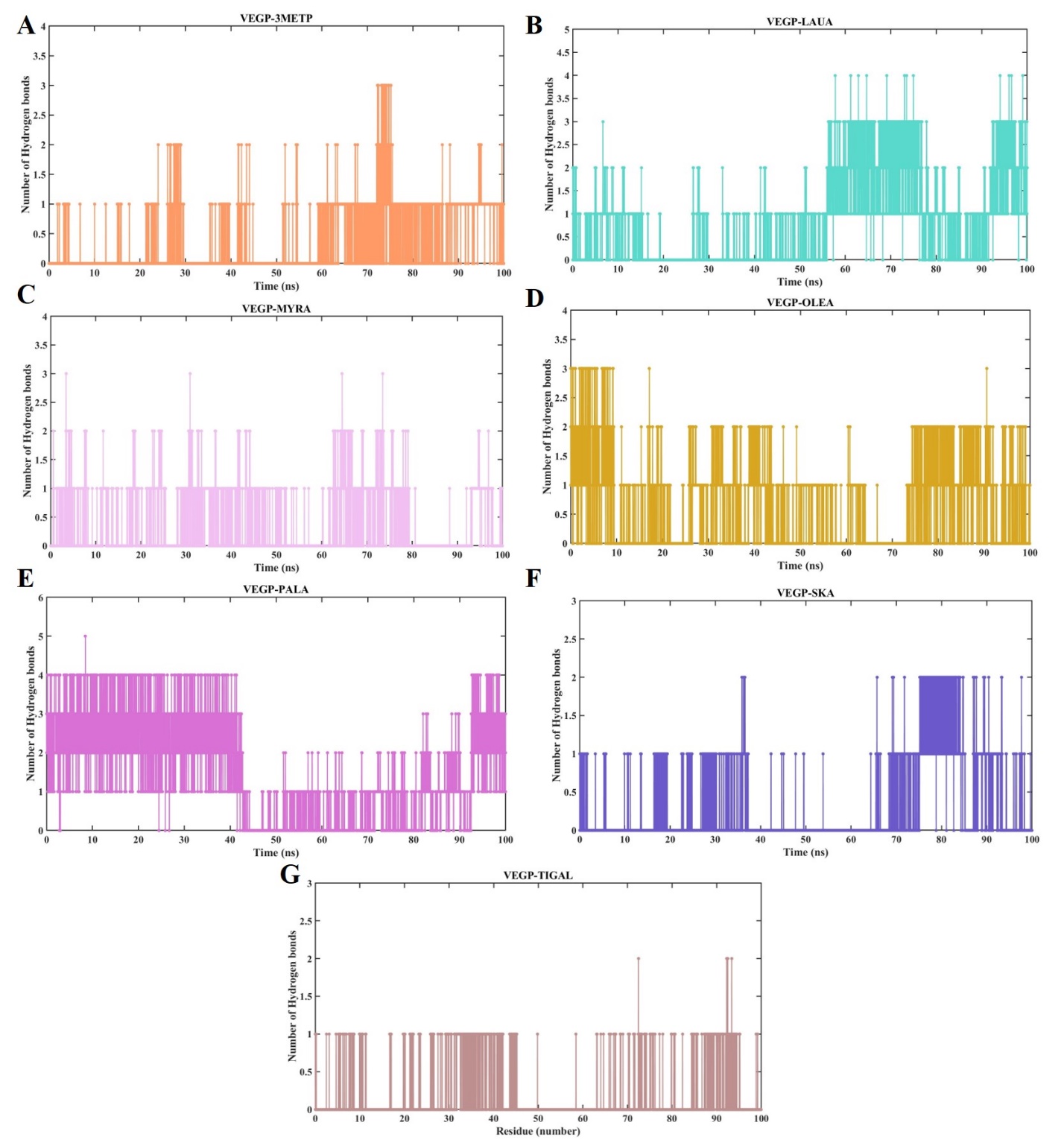


**Supplementary Figure 6:** Number of Intermolecular hydrogen bonds between compounds [3-Methylphenol (Peach), Lauric Acid (Aquamarine), Myristic Acid (Lavender Blush), Oleic Acid (Goldenrod), Palmitic Acid (Orchid), Skatole (Slate Blue), and Tiglic aldehyde (Rosy Brown)] and VEGP from MDS trajectory.


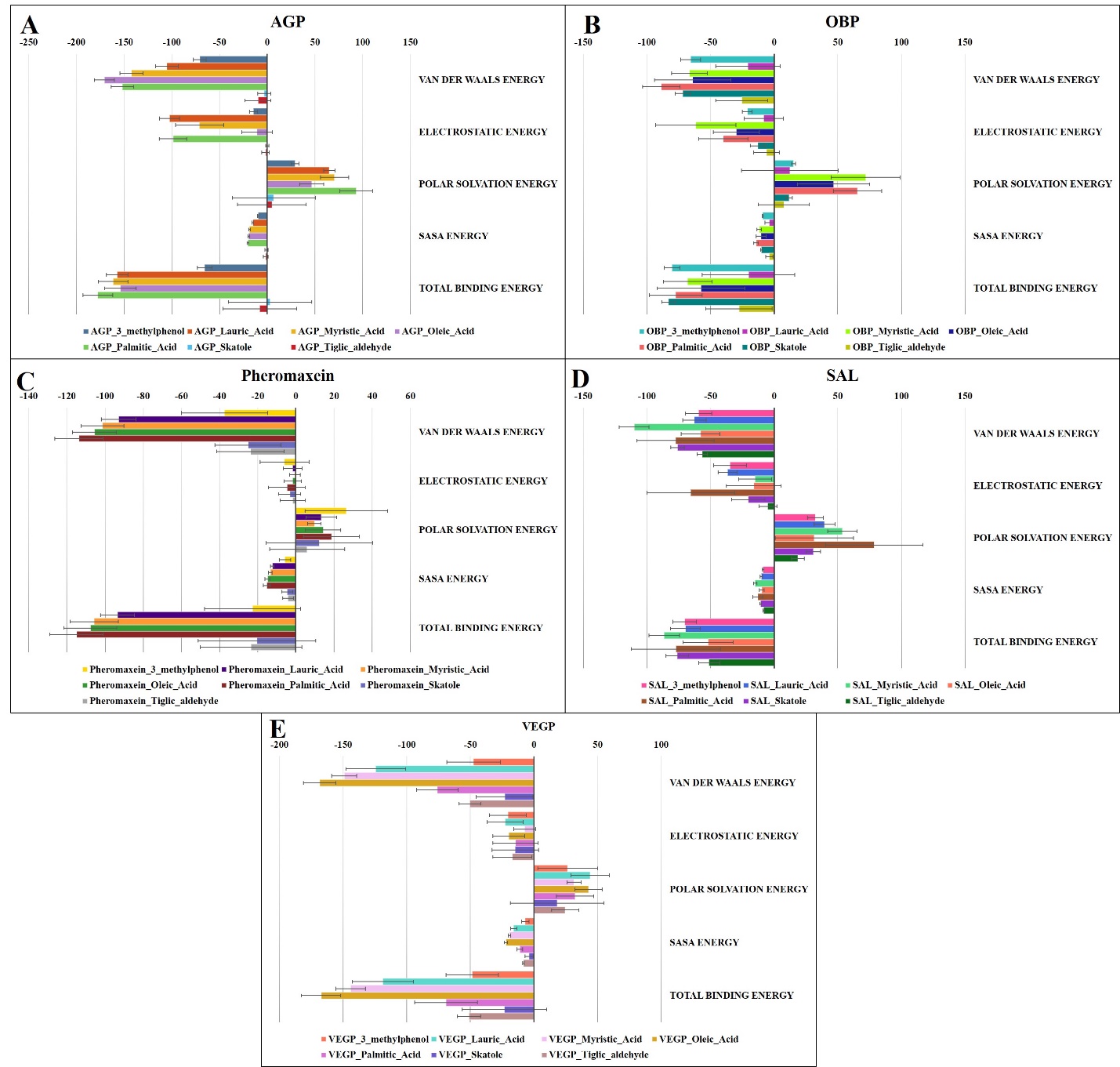


**Supplementary Figure 7:** Decomposition analysis of the components contributing to the total binding energy of all the complexes was determined using MM/PBSA binding free energy calculations. The bars in the plots indicate the standard error of the average. Energy values in the plots are represented in kJ/mol. (A) RMSD probability distribution plot of AGP-3-Methylphenol (Blue-Gray), AGP-Lauric Acid (Burnt Orange), AGP-Myristic Acid (Mustard Yellow), AGP-Oleic Acid (Lavender), AGP-Palmitic Acid (Seafoam Green), AGP-Skatole (Sky Blue), and AGP-Tiglic aldehyde (Brick Red). (B) RMSD probability distribution plot of OBP-3-Methylphenol (Turquoise), OBP-Lauric Acid (Deep Magenta), OBP-Myristic Acid (Lime), OBP-Oleic Acid (Navy Blue), OBP-Palmitic Acid (Salmon pink), OBP-Skatole (Teal Green), and OBP-Tiglic aldehyde (yellow-green) (C) RMSD probability distribution plot of Pheromaxein-3-Methylphenol (Gold), Pheromaxein-Lauric Acid (Indigo), Pheromaxein-Myristic Acid (Tangerine), Pheromaxein-Oleic Acid (Forest Green), Pheromaxein-Palmitic Acid (Dark Red), Pheromaxein-Skatole (Pale Blue), and Pheromaxein-Tiglic aldehyde (Silver Gray) (D) RMSD probability distribution plot of SAL-3-Methylphenol (Hot Pink), SAL-Lauric Acid (Royal Blue), SAL-Myristic Acid (Mint Green), SAL-Oleic Acid (Tomato), SAL-Palmitic Acid (Sienna Brown), SAL-Skatole (Violet), and SAL-Tiglic aldehyde (Deep green) (E) RMSD probability distribution plot of VEGP-3-Methylphenol (Peach), VEGP-Lauric Acid (Aquamarine), VEGP-Myristic Acid (Lavender Blush), VEGP-Oleic Acid (Goldenrod), VEGP-Palmitic Acid (Orchid), VEGP-Skatole (Slate Blue), and VEGP-Tiglic aldehyde (Rosy Brown).

**Supplementary Table 1:** The Two-Dimensional (2D) structure of all the Ligands was downloaded from PubChem.

| **S.No** | **Ligand** | **2D structure** |
| --- | --- | --- |
| 1 | 3-methylphenol | 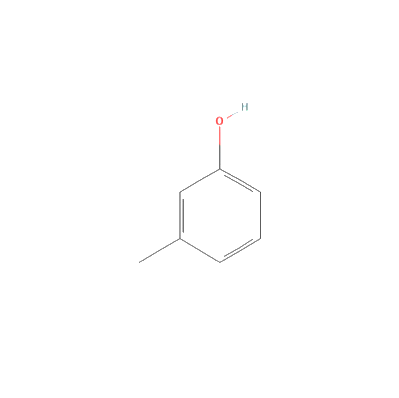 |
| 2 | Lauric Acid | 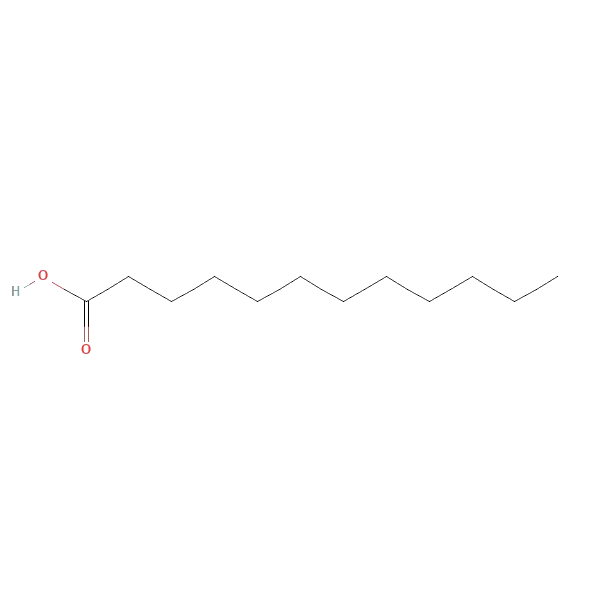 |
| 3 | Myristic Acid | 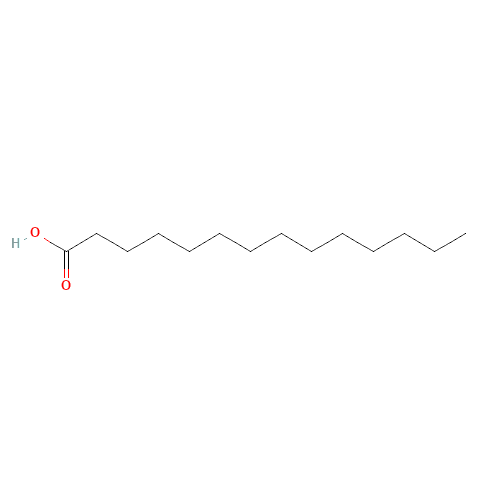 |
| 4 | Oleic Acid | 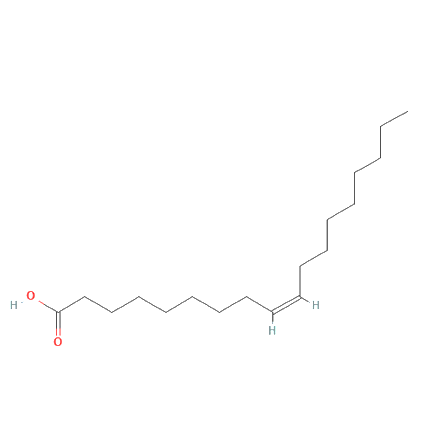 |
| 5 | Palmitic Acid | 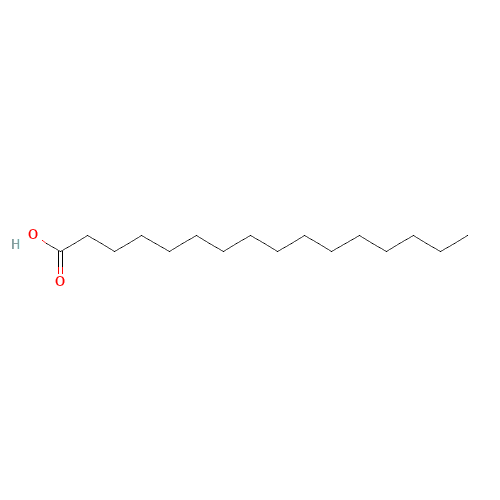 |
| 6 | Skatole | 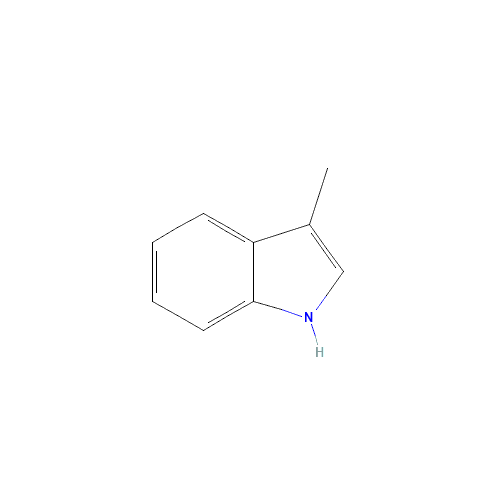 |
| 7 | Tiglic Aldehyde | 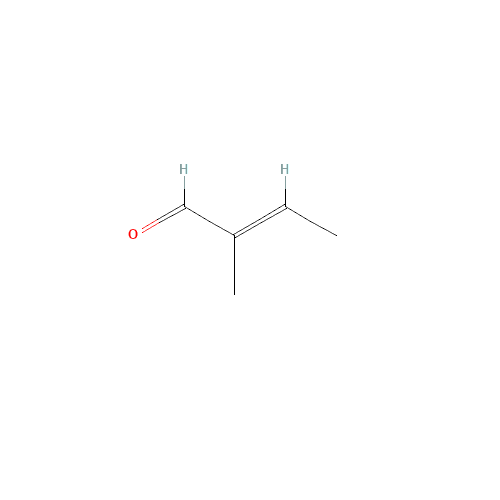 |

**Supplementary Table 2:** The Three-Dimensional (3D) structure and active site of all the proteins. The active site regions of each protein structure were highlighted in red color.

| **S.No** | **Protein** | **3D structure and active site of the protein** | **Key residues in the binding pocket predicted by CASTp** |
| --- | --- | --- | --- |
| 1 | AGP | 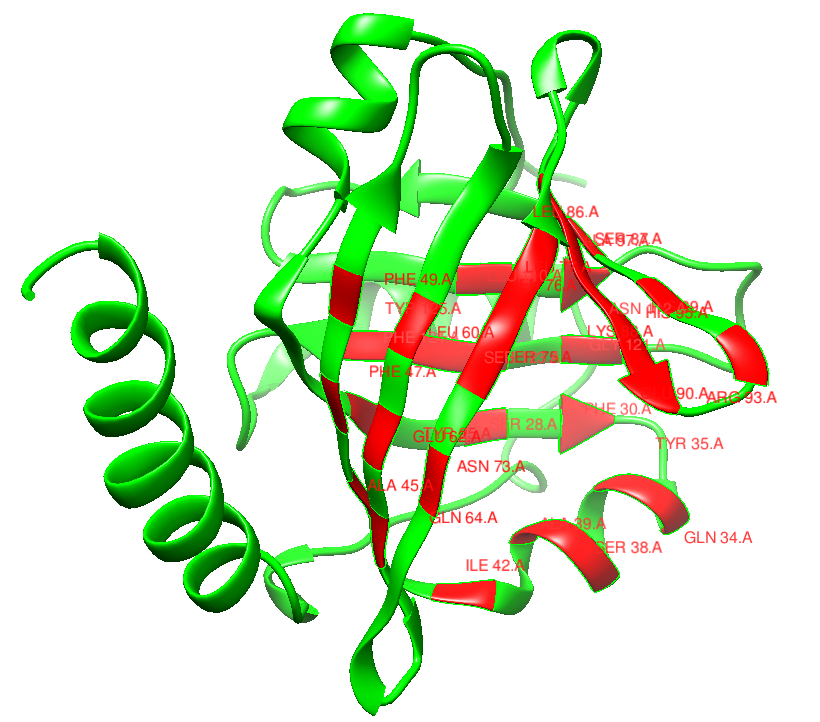 | TYR25, SER28, PHE30, GLN34, TYR35, SER38, ALA39, ILE42, ALA45, PHE47, PHE49, LEU60, GLU62, GLN64, ASN73, SER75, SER76, LEU77, LEU86, SER87, LYS88, HIS89, GLU90, ARG93, HIS95, ALA97, LEU110, ASN112, GLY121, SER123, PHE124, and TYR125. |
| 2 | OBP | 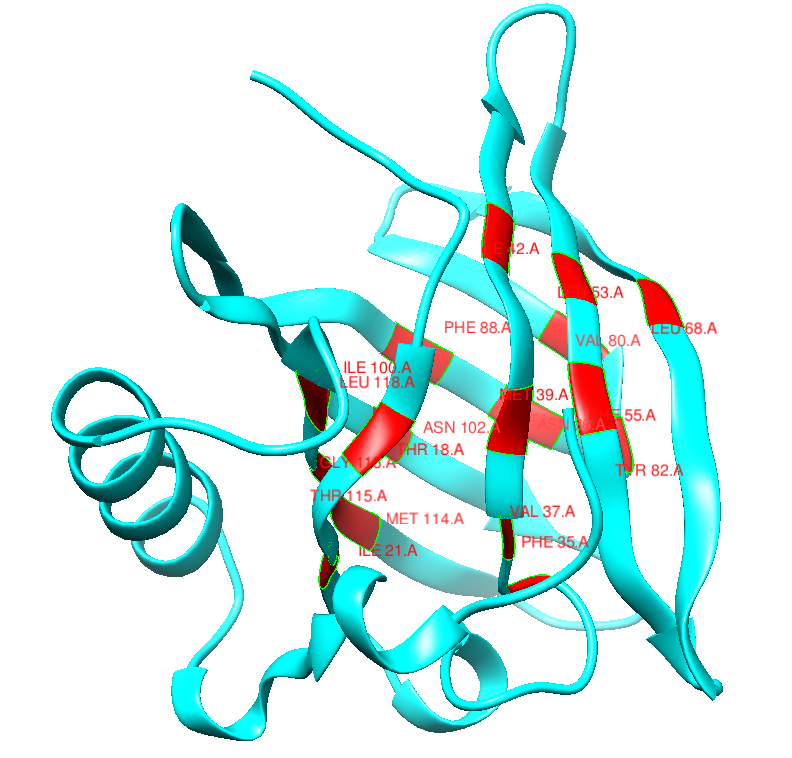 | THR18, ILE21, PHE35, VAL37, MET39, ILE42, LEU53, PHE55, LEU68, VAL80, TYR82, ASN86, PHE88, ILE100, ASN102, MET114, THR115, GLY116, and LEU118. |
| 3 | Pheromaxein | 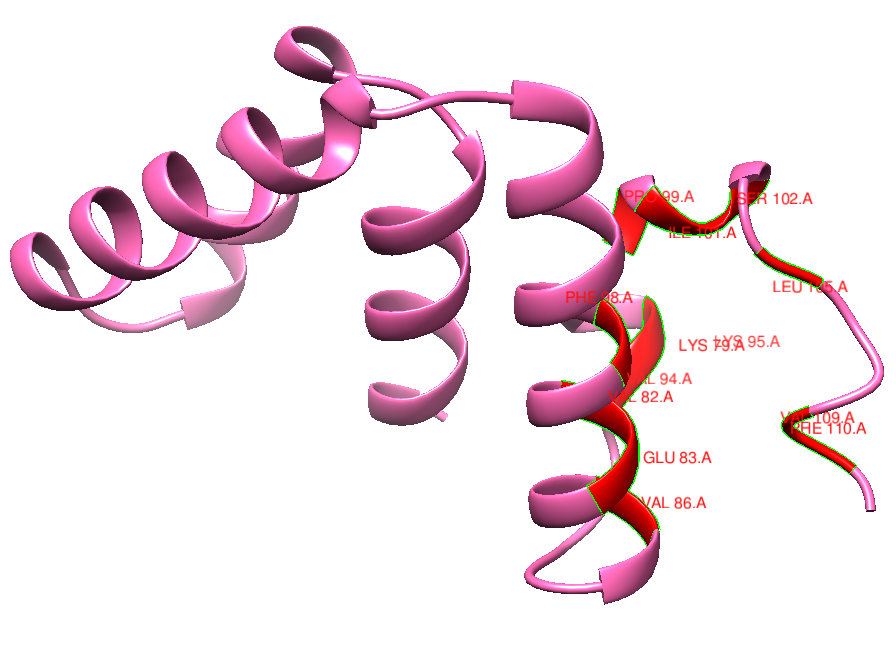 | LYS79, VAL82, GLU83, VAL86, VAL94, LYS95, PHE98, PRO99, ILE101, SER102, LEU105, VAL109, and PHE110. |
| 4 | SAL | 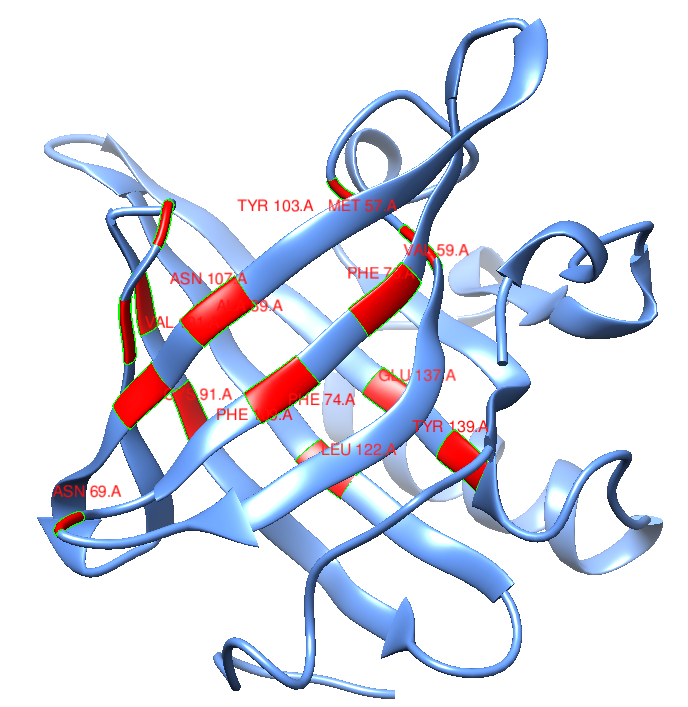 | MET57, VAL59, VAL69, PHE74, PHE76, ALA89, CYS91, VAL101, TYR103, ASN107, PHE109, LEU122, GLU137, and TYR139. |
| 5 | VEGP | 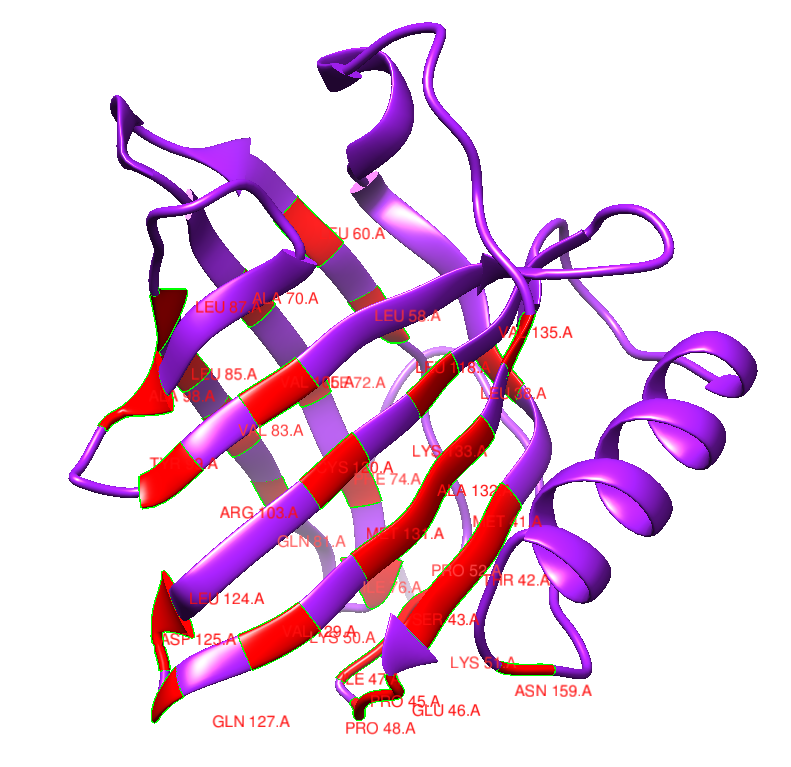 | LEU38, MET41, THR42, SER43, PRO45, GLU46, ILE47, PRO48, LYS50, LYS51, PRO52, LEU58, LEU60, ALA70, ILE72, PHE74, ILE76, GLN81, VAL83, LEU85, LEU87, ALA98, TYR99, ARG103, VAL105, LEU118, CYS120, LEU124, ASP125, GLN127, VAL129, MET131, ALA132, LYS133, VAL135, ASN159. |

**Supplementary Table 3:** The grid box's coordinates and dimensions for each structure utilized in AutoDock Vina are listed.

| **S.No** | **Protein** | **Ligands** | **Center (Å)** | | | **Box dimension (Å)** | | |
| --- | --- | --- | --- | --- | --- | --- | --- | --- |
|  |  |  | **X** | **y** | **z** | **x** | **y** | **z** |
| 1 | AGP | 3-Methylphenol, Lauric Acid, Myristic Acid, Oleic Acid, Palmitic Acid, Skatole, Tiglic Aldehyde | 63.282 | 56.764 | 58.399 | 40 | 40 | 40 |
| 2 | OBP | 3-Methylphenol, Lauric Acid, Myristic Acid, Oleic Acid, Palmitic Acid, Skatole, Tiglic Aldehyde | 2.594 | 26.311 | 11.859 | 72 | 70 | 92 |
| 3 | Pheromaxein | 3-Methylphenol, Lauric Acid, Myristic Acid, Oleic Acid, Palmitic Acid, Skatole, Tiglic Aldehyde | 8.686 | 11.408 | 5.553 | 72 | 70 | 92 |
| 4 | SAL | 3-Methylphenol, Lauric Acid, Myristic Acid, Oleic Acid, Palmitic Acid, Skatole, Tiglic Aldehyde | 7.931 | 20.782 | 19.56 | 72 | 70 | 92 |
| 5 | VEGP | 3-Methylphenol, Lauric Acid, Myristic Acid, Oleic Acid, Palmitic Acid, Skatole, Tiglic Aldehyde | -0.491 | -1.723 | -1.029 | 90 | 96 | 92 |

**Supplementary Table 4:** The calculated Gibbs Free Energy of Binding (ΔG) for each protein-ligand pair. In this equation, ΔH is the change in enthalpy (kJ/mol), and TΔS is the Temperature (K) multiplied by the change in entropy (J/mol K) and converted into (kJ/mol).

| **Complexes** | **ΔH (kJ/mol)** | **-TΔS (kJ/mol)** | **ΔG = ΔH – TΔS**  **(kJ/mol)** |
| --- | --- | --- | --- |
| AGP-3-Methylphenol | -65.573 | -320.655 | -386.228 |
| AGP-Lauric Acid | -157.312 | -301.155 | -458.467 |
| AGP-Myristic Acid | -161.364 | -320.517 | -481.881 |
| AGP-Oleic Acid | -153.944 | -304.062 | -458.006 |
| AGP-Palmitic Acid | -177.532 | -310.497 | -488.029 |
| AGP-Skatole | 2.947 | -300.813 | -297.866 |
| AGP-Tiglic aldehyde | -8.001 | -311.307 | -319.308 |
| OBP-3-Methylphenol | -80.378 | -263.507 | -343.885 |
| OBP-Lauric Acid | -20.173 | -272.271 | -292.444 |
| OBP-Myristic Acid | -68.021 | -267.919 | -335.941 |
| OBP-Oleic Acid | -57.490 | -281.221 | -338.712 |
| OBP-Palmitic Acid | -77.451 | -273.535 | -350.986 |
| OBP-Skatole | -83.004 | -269.859 | -352.863 |
| OBP-Tiglic aldehyde | -27.613 | -273.059 | -300.671 |
| Pheromaxein-3-Methylphenol | -22.700 | -260.467 | -283.167 |
| Pheromaxein-Lauric Acid | -93.500 | -264.443 | -357.943 |
| Pheromaxein-Myristic Acid | -105.631 | -252.690 | -358.321 |
| Pheromaxein-Oleic Acid | -107.693 | -236.543 | -344.236 |
| Pheromaxein-Palmitic Acid | -114.967 | -270.713 | -385.680 |
| Pheromaxein-Skatole | -20.420 | -252.883 | -273.303 |
| Pheromaxein-Tiglic aldehyde | -23.563 | -271.205 | -294.768 |
| SAL-3-Methylphenol | -70.448 | -273.232 | -343.680 |
| SAL-Lauric Acid | -69.813 | -268.647 | -338.461 |
| SAL-Myristic Acid | -86.449 | -278.470 | -364.919 |
| SAL-Oleic Acid | -51.935 | -284.226 | -336.161 |
| SAL-Palmitic Acid | -77.193 | -283.390 | -360.583 |
| SAL-Skatole | -76.113 | -274.350 | -350.463 |
| SAL-Tiglic aldehyde | -51.237 | -281.015 | -332.237 |
| VEGP-3-Methylphenol | -48.541 | -298.907 | -347.448 |
| VEGP-Lauric Acid | -118.807 | -288.452 | -407.259 |
| VEGP-Myristic Acid | -144.099 | -304.608 | -448.707 |
| VEGP-Oleic Acid | -167.288 | -317.526 | -484.814 |
| VEGP-Palmitic Acid | -69.120 | -320.613 | -389.733 |
| VEGP-Skatole | -23.293 | -301.149 | -324.442 |
| VEGP-Tiglic aldehyde | -51.025 | -303.147 | -354.172 |
